# Comprehensive analysis of the prognosis and immune infiltration of TMC family members in renal clear cell carcinoma

**DOI:** 10.1038/s41598-023-38914-z

**Published:** 2023-07-19

**Authors:** Wenbin Tang, Zhiyuan Shi, Yasheng Zhu, Zhengda Shan, Aimin Jiang, Anbang Wang, Ming Chen, Yi Bao, Guanqun Ju, Weidong Xu, Junkai Wang

**Affiliations:** 1 Department of Urology, Changzheng Hospital, Naval Medical University, NO. 415 Fengyang Road, Shanghai, 200003 China; 2 Department of Urology, School of Medicine, Xiang’an Hospital of Xiamen University, Xiamen University, NO. 4221 Xiang’an South Road, Xiamen, 361101 Fujian Province China; 3 Department of Urology, Changhai Hospital, Naval Medical University, NO. 168 Changhai Road, Shanghai, 200082 China; 4 School of Medicine, Sun Yat-Sen University, NO. 66 Gongchang Road, Shenzhen, 518107 Guangdong Province China

**Keywords:** Urological cancer, Data mining

## Abstract

Renal cancer is a common malignancy of the urinary system, and renal clear cell carcinoma (RCCC) is the most common pathological type. Transmembrane channel-like (TMC) protein is an evolutionarily conserved gene family containing 8 members, however there is still a lack of comprehensive analysis about TMC family members in RCCC. In this study, we analyzed the expression of TMC family members in RCCC from TCGA and investigated the prognosis values and immune infiltration of TMC family members in RCCC. We found that TMC2, TMC3, TMC5, TMC7 and TMC8 were significantly related with overall survival (OS) of RCCC patients. TMC3, TMC6, and TMC8 was positively correlated with the degree of immune infiltration in RCCC. TMC2, TMC6, TMC7, and TMC8 were positively correlated with immune checkpoint genes, whereas TMC4 was negative. According to KEGG and GO analysis, almost all TMCs except TMC4 were involved in the immune response. Thus, we may regard the TMC family members as novel biomarkers to predict potential prognosis and immunotherapeutic response in RCCC patients.

## Introduction

Renal cancer is one of the major urological malignancies. According to the novel global cancer statistics in 2020, there were more than 430,000 new cases and about 180,000 deaths of renal cancer^1^. In the United States, there were nearly 79,000 new renal cancer patients and 13,920 deaths in 2022^2^. Renal cell carcinoma (RCC) is the most common pathological type of renal cancer^3^. There are many histological subtypes of RCC, and renal clear cell carcinoma (RCCC) accounts for nearly 85% of RCC^4^. Early diagnosis and treatment can improve the prognosis and prolong the survival of RCCC patients. However, the clinical symptoms of early RCCC are not obvious, and more than 60% patients are accidentally found during routine physical examination^5^. 30% patients were discovered distant metastases at the initial diagnosis, and approximately 30% patients with radical nephrectomy treatment eventually developed metastatic renal cancer^6^.

The existence of immune infiltration in tumor tissue was recognized in 1922^7^, and Hamlin first demonstrated the relationship between lymphocytic infiltration and prognosis in breast cancer^8^. Another study showed that the degree of lymphocyte infiltration in primary tumors positively correlated with distant metastasis, and T lymphocyte was the largest and most important component^9^. RCCC is not sensitive to chemotherapy or radiotherapy^10,11^, and the occasional spontaneous tumor regression and the presence of tumor-infiltrating immune cells indicated that adaptive immunity might play an crucial role in RCC^12^. Over the past few decades, immunotherapy with high-dose IL-2 or interferon-α has been applied for advanced RCC treatment, whereas the response rate to immunotherapy only ranged between 15 and 25%^13^. In recent years, various immune checkpoint inhibitors have been approved for RCCC treatment, which bring a novel strategy for immunotherapy, especially targeting programmed cell death protein-1 (PD-1) and programmed cell death ligand-1(PD-L1). PD-1 is a molecule that is expressed on the surface of T lymphocytes and combines with its ligand PD-L1 on a variety of tumor cells. This interaction could induce T lymphocyte apoptosis and eventually lead to the immune escape of tumor cells^14^. An early study demonstrated that RCCC tumors possess various level of PD-L1 expression^15^, and patients with tumors expressing PD-L1 had significantly lower 5-year cancer-specific survival (CSS) than those without PD-L1^16^. Besides, the expression of PD-L1 on metastatic RCC cells is directly related to aggressive pathologic features^17^.

Transmembrane channel-like (TMC) protein is a group of evolutionarily conserved ion channel-like membrane proteins, which contained TMC1, TMC2, TMC3, TMC4, TMC5, TMC6, TMC7, and TMC8. Based on the nucleotide sequence similarity, the TMC proteins in mammalian could be divided into three subfamilies: subfamily A(TMC1, 2 and 3), subfamily B (TMC5 and 6), and subfamily C (TMC4, 7 and 8)^18^. As conserved membrane proteins, TMC proteins are widely involved in sensorimotor functions in various species, such as hearing and food texture detection. Kurima et al. first reported that TMC1 and TMC2 were members of a gene family encoding transmembrane proteins and associated with deafness in 2002^19^. In 2003, Kurima et al. described six additional TMC paralogs(TMC3 to TMC8) in humans and mice and homologs in other species (20).TMC1 and TMC2 are both expressed in the stereocilia of mature vestibular hair cells^21,22^, and their mutation could lead to hearing loss^23^. TMC3 can negatively regulate the expression of interleukin-10 in macrophages and intestinal epithelial cells and plays a vital role in innate immune response^24^. TMC4 is a chloride channel involved in high-concentration salt taste transduction, expressed in taste bud cells of the posterior tongue^25^. Besides, its variation increases the risk of nonalcoholic fatty liver disease^26^. The expression level of TMC5 is related to the poor outcomes of squamous cell carcinoma, acute myeloid leukemia, and prostate cancer^27,28^. In recent years, there has been evidence that TMC6 and TMC8 play a role in cervical cancer and squamous cell carcinoma^29,30^. In the meantime, Cheng Y et al. identified TMC7 as a potential prognostic biomarker for pancreatic cancer^31^.

TMC members play an essential role in development and progress of several diseases, such as the prognosis and immune infiltration of human cancers^32^, however the role of TMC family members of RCCC has not been investigated. In this study, we analyzed this and found that it is highly possible and convenient to study the underlying occurrence and development mechanism of RCCC, explore new and therapeutic methods, and improve the survival rate of RCCC patients.

## Results

### TMC family expression in RCCC and normal tissues

**Figure 1:**
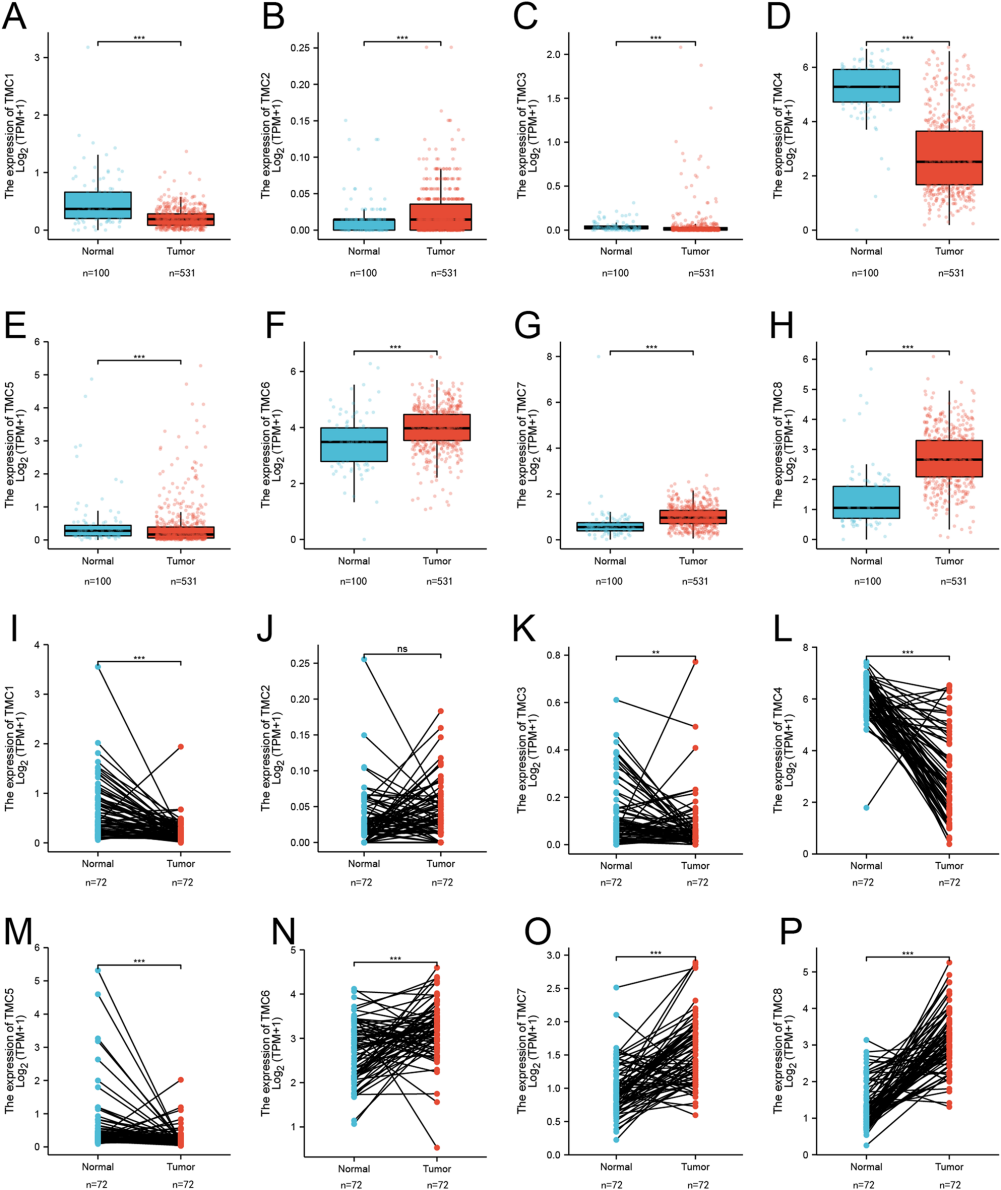
The expression of TMCs in normal renal and RCCC tissues. The graphs are generated using R software. **p < 0.01, ***p < 0.001, ns: no statistically significant. (**A**–**H**) TMC family expression in unpaired normal renal and RCCC tissues. (**I**–**P**) TMC family expression in paired normal renal and RCCC tissues.

As displayed in Fig. 1A,B,C,D,E,F,G,H, TMC2, TMC6, TMC7, and TMC8 were upregulated in RCCC tissues compared with normal, while TMC1, TMC3, TMC4, and TMC5 was downregulated. Subsequently, we used the same method to evaluate TMC gene family expression in 72 pairs of RCCC and adjacent samples. The results demonstrated there was no significant difference in TMC2 expression between tumor and normal tissues, and expression of other TMCs was consistent with that before (Fig. 1I,J,K,L,M,N,O,P).

**Figure 2:**
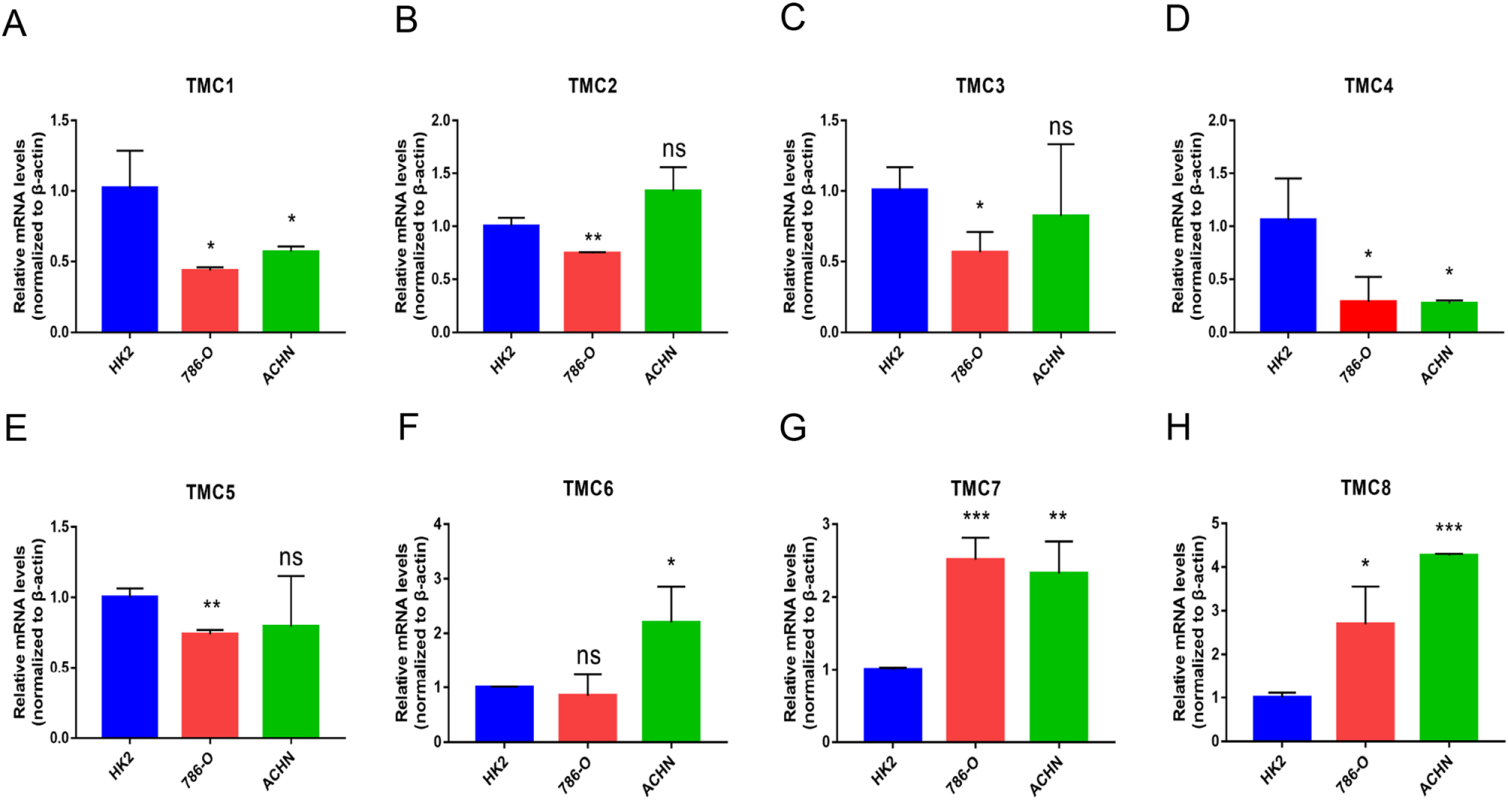
The expression of the TMC family genes in RCCC compared with the normal. (**A**–**H**) TMCs’ mRNA levels in RCCC cell lines quantified by Real-time PCR. *p < 0.05, **p < 0.01, ***p < 0.001, ns: no statistically significant.

In addition, we utilized the UALCAN and GEPIA2 databases to further contrast the expression levels of TMC1–TMC8 in RCCC and normal renal tissues. The results of the two online databases are roughly the same as those obtained by R software (Figure S1). And we examined the difference in TMC family expression between normal renal cortex proximal convoluted tubule epithelial cell line and clear cell renal cell carcinoma cell lines. Compared with normal renal cell line, TMC7 and TMC8 in RCCC cell lines (786-O and ACHN) significantly upregulated, while the expressions of TMC1 and TMC4 were decreased (Fig. 2A,B,C,D,E,F,G,H).

### Role of the TMC family in the survival of RCCC patients

**Figure 3:**
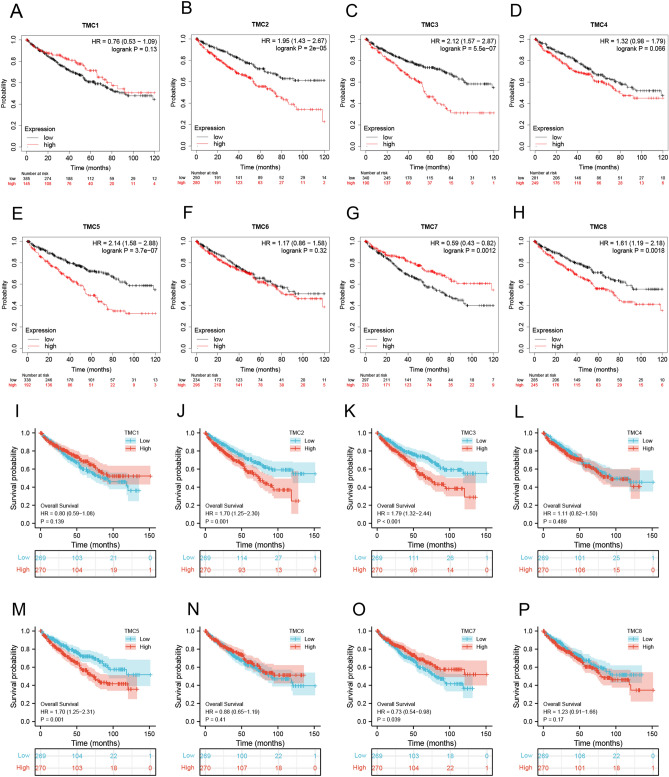
The prognostic significance of TMC family expression in RCCC patients. (**A**–**H**) Correlation between TMC family expression and OS of RCCC patients from the KM plotter database. (**I**–**P**) Correlation between TMC family expression and OS of RCCC patients using R software.

We used the online Kaplan- Meier Plotter tool to analyze the relationship between TMCs and Overall Survival (OS) in RCCC patients. As shown in Fig. 3A,B,C,D,E,F,G,H, high expression of TMC2, TMC3, TMC5, and TMC8 and low TMC7 indicated a worser survival time of RCCC patients.

In addition, we utilized R software to verify the correlation between the expression of TMC family members and the survival of RCCC patients. Our results illustrated patients with a high level of TMC2, TMC3, and TMC4 and those with a low TMC7 had a shorter OS (Fig. 3I,J,K,L,M,N,O,P). High expression of TMC2, TMC3, and TMC5 and low TMC7 negatively correlated with the Disease-Specific Survival (DSS) of RCCC patients (Figure S2A,B,C,D,E,F,G,H). And the increased expression of TMC3 and TMC5 and low TMC6 and TMC7 was negatively correlated with the Progress Free Interval (PFI) of RCCC patients (Figure S2I,J,K,L,M,N,O,P).

Univariate COX analysis and multivariate COX analysis were performed to explore the role of TMCs in the survival of RCCC patients. Results of the univariate COX regression model indicated the high levels of TMC2, TMC3, and TMC5 were risk factors (Hazard ratio > 1, *p* < 0.001), and high TMC7 expression was a protective factor (Hazard ratio < 1, *p* < 0.05). However, multivariate COX analysis showed that only TMC2 was an independent prognostic factor (Table 1).

**Table 1:** Univariate analysis and multivariate analysis of TMCs.

Characteristics	Total (N)	Univariate analysis		Multivariate analysis	
		Hazard ratio (95% CI)	*P* value	Hazard ratio (95% CI)	*P* value
TMC1	539				
Low	269	Reference			
High	270	0.797 (0.590–1.076)	0.139		
TMC2	539				
Low	269	Reference			
High	270	1.696 (1.250–2.300)	< 0.001	1.950 (1.241–3.062)	0.004
TMC3	539				
Low	269	Reference			
High	270	1.792 (1.317–2.438)	< 0.001	1.320 (0.856–2.036)	0.209
TMC4	539				
Low	269	Reference			
High	270	1.111 (0.824–1.498)	0.489		
TMC5	539				
Low	269	Reference			
High	270	1.696 (1.247–2.308)	< 0.001	1.307 (0.850–2.009)	0.222
TMC6	539				
Low	269	Reference			
High	270	0.882 (0.653–1.190)	0.410		
TMC7	539				
Low	269	Reference			
High	270	0.727 (0.537–0.984)	0.039	0.912 (0.595–1.397)	0.671
TMC8	539				
Low	269	Reference			
High	270	1.233 (0.914–1.663)	0.170		

### TMC family was related to histologic grade and pathologic stage of RCCC

**Figure 4:**
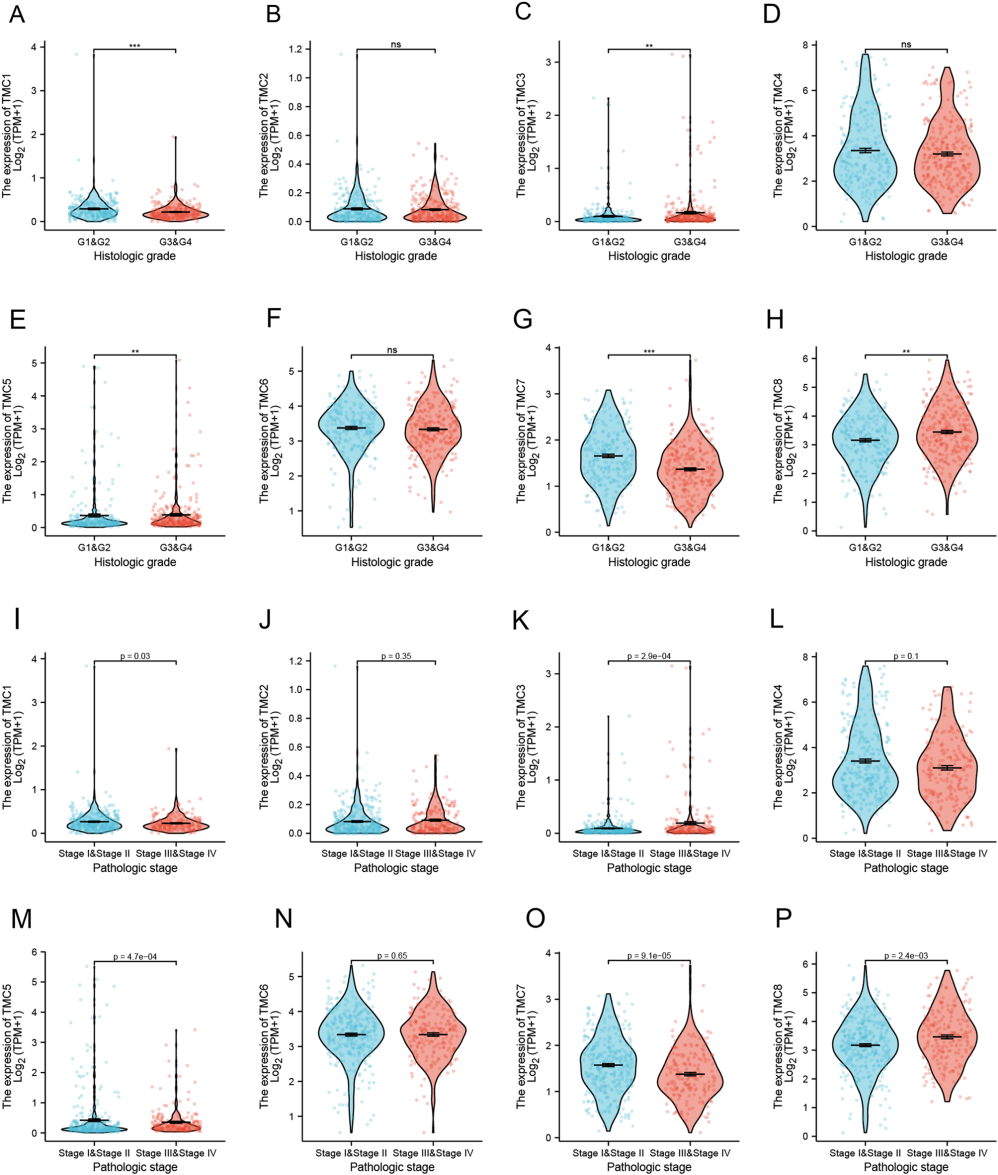
Correlation analysis between TMC family expression and clinicopathologic features in RCCC. (**A**–**H**) Correlation analysis between TMC family expression and histologic grade of RCCC patients. (**I**–**P**) Correlation analysis between TMC family expression and pathologic stage of RCCC patients. **p < 0.01, ***p < 0.001, ns: no statistically significant.

We divided RCCC patients into two cohorts according to the tumor's histological grade (Fuhrman grading system) or pathological stage and investigated the TMC family expression differences using R software. As it turned out in Fig. 4A,B,C,D,E,F,G,H, the expressions of TMC1, TMC3, TMC5, TMC7, and TMC8 significantly differed between low and high histological grades of RCCC (Grade I & II vs. Grade III & IV). As we expected, TMC1, TMC3, TMC5, TMC7, and TMC8 also showed the same trend between early and late pathological stages (Stage I & II vs. Stage III & IV) as the change in histological grade cohorts (Fig. 4I,J,K,L,M,N,O,P). And then, the "pathological stage map" module of the GEPIA2 website was used to further confirm the relationship between the expression of TMCs and the pathological staging of RCCC. Compared with the R software results, the TMC1, TMC3, TMC7, and TMC8 showed significant pathological stage-specific changes except for TMC5 (Figure S3A,B,C,D,E,F,G,H).

ROC analysis was performed to comprehensively evaluate the role of TMC family expression in the prognostic outcome of RCCC patients. In the ability to predict normal and tumor outcomes, the predictive ability of TMC2, TMC3, TMC5, and TMC6 has low accuracy (AUC between 0.5 and 0.7). The ability of TMC1, TMC7, TMC8 had a certain accuracy (AUC between 0.7 and 0.9), and TMC4 had the highest accuracy (AUC = 0.903, CI = 0.870–0.936) as showed in the Figure S3I,J,K,L,M,N,O,P.

### TMC family was associated with immune infiltration in RCCC

The tumor microenvironment (TME) is composed of tumor cells, recruited cells (e.g., immune cells, vascular endothelial cells, and stromal cells), secreted products of corresponding cells (such as cytokines and chemokines), and non-cellular components of the extracellular matrix. Tumor-associated immune cell infiltration is an integral component of TME and is closely related to the prognosis and lymph node metastasis of malignant tumors.

Our study found that TMC2, TMC6, TMC7, and TMC8 upregulated, while TMC1, TMC3, TMC4, and TMC5 downregulated in RCCC tissues. It seemed unreasonable that RCCC patients with high TMC2, TMC3, TMC4, TMC5, and TMC8 and those with low TMC6 and TMC7 had a shorter survival time. But RNA-seq is a bulk-seq in which a variety of cells intermingled. Genes that RNA-seq highly expresses in tumor tissue are likely to be molecules in immune cells, depending on the immune infiltration of the tumor.

**Figure 5:**
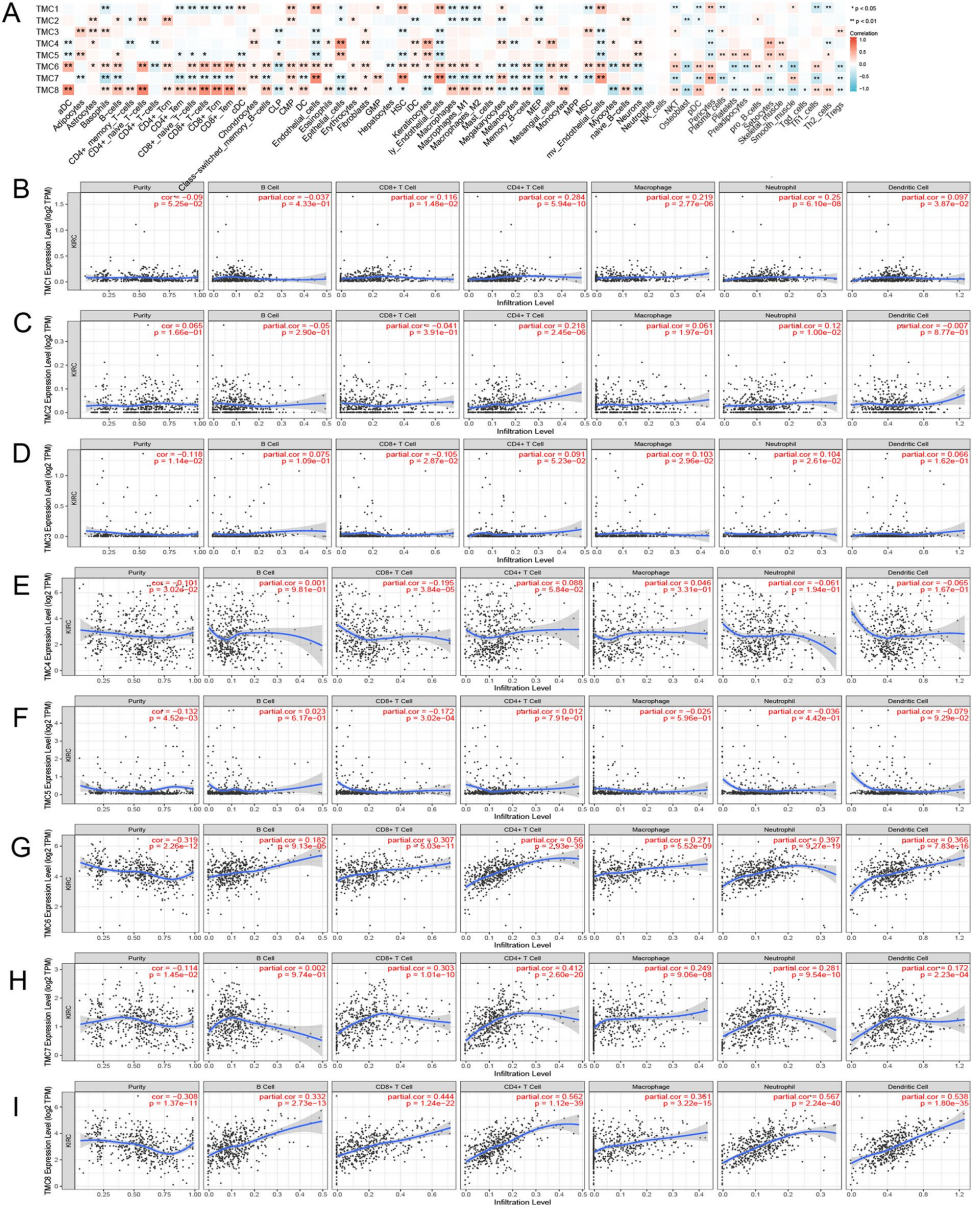
Relationship between TMC family expression and immune infiltrating cells in RCCC. (**A**) Correlation between TMC family expression and levels of immune infiltration cells based on R software. *p < 0.05, **p < 0.01. (**B**–**I**) Correlation between TMC family expression and level of immune infiltration cells based on TIMER database.

**Figure 6:**
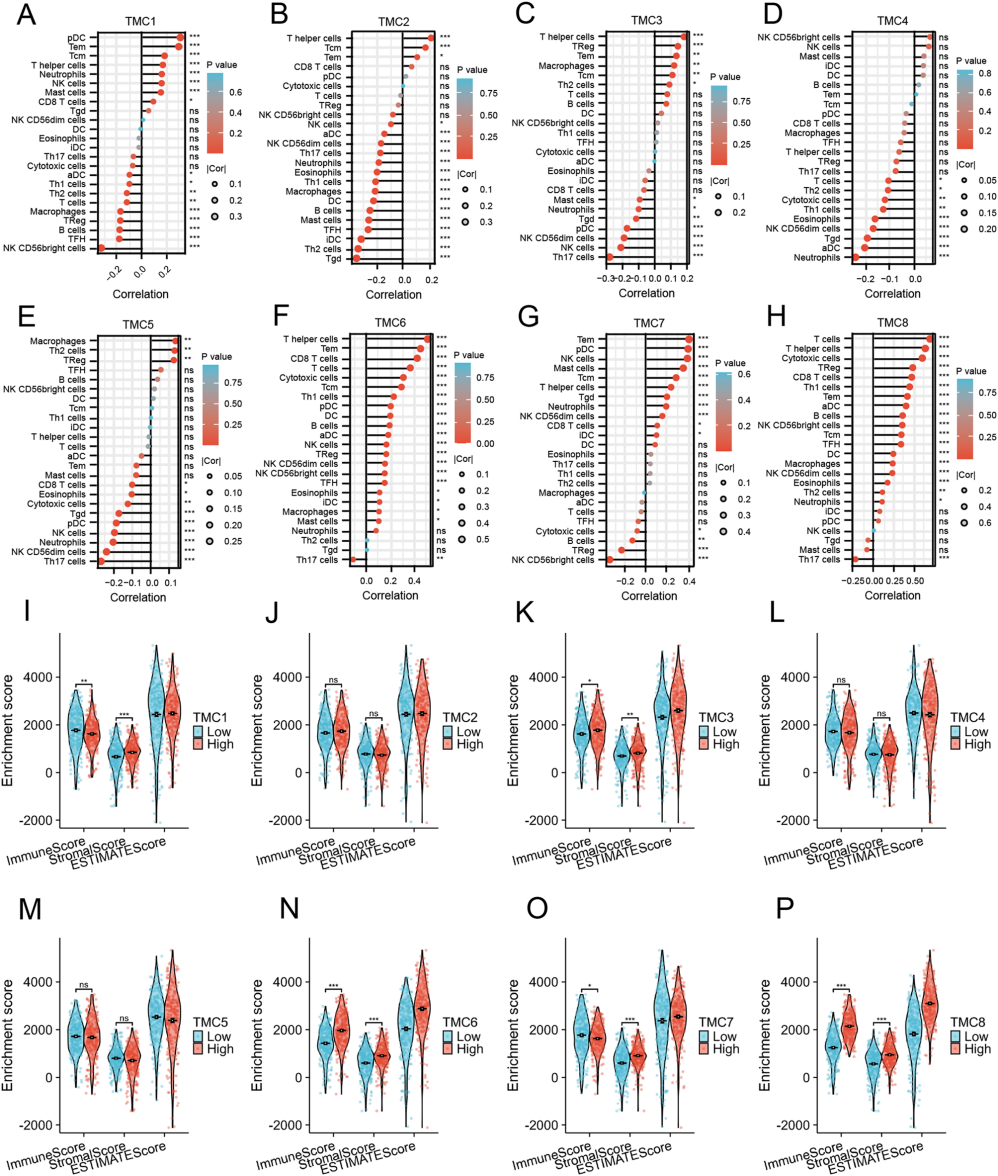
Relationship between TMC family expression and immune infiltration in RCCC. *p < 0.05, **p < 0.01, ***p < 0.001, ns: no statistically significant. (**A**–**H**) Lollipop plot indicating the relationship between TMC family expression and immune infiltration cells. (**I**–**P**) Violin plot showing the relationship between TMC expression and the level of immune infiltration in RCCC based on R package “estimate”.

According to the available data, we calculated 64 immune cells in RCCC using the "xCELL" algorithm using R software. Interestingly, the expression of TMC2, TMC5, TMC6, and TMC8 positively correlated with the number of B cells, while TMC7 negatively correlated with B cells. TMC4 and TMC6 negatively correlated with the degree of CD4 + T cells. The expression of TMC6 and TMC8 positively correlated with the number of CD8 + T cells, while TMC1, TMC5, and TMC7 negatively correlated with CD8 + T cells. The expression of TMC3, TMC5, TMC6, and TMC8 positively correlated with the number of NKT cells, while TMC7 negatively correlated with NKT cells. TMC6 and TMC8 positively correlated with the degree of macrophages, while TMC1, TMC2, and TMC7 negatively correlated with macrophages. The expression of TMC6 and TMC8 positively correlated with the number of dendritic cells, while TMC7 negatively correlated with dendritic cells (Fig. 5A). Next, we utilized the “ssGSEA” algorithm and TIMER2.0 to further verify the influence of TMC family genes on the immune state of RCCC and obtained roughly the same results (Fig. 5B,C,D,E,F,G,H,I and 6A,B,C,D,E,F,G,H).

The two major non-tumor components of TME, considered predictive biomarkers in cancer patients, are immune and stromal cells. A higher score, as calculated using ImmuneScore or StromalScore, indicates a more significant proportion of immune or stromal components in the TME. ESTIMATEScore is the sum of ImmuneScore and StromalScore, and thus represents the combined ratio of these two components in the TME. The results with the R package “estimate” hint that the high expression of TMC3, TMC6, and TMC8 positively correlated with the degree of immune infiltration in RCCC (Fig. 6I,J,K,L,M,N,O,P).

**Figure 7:**
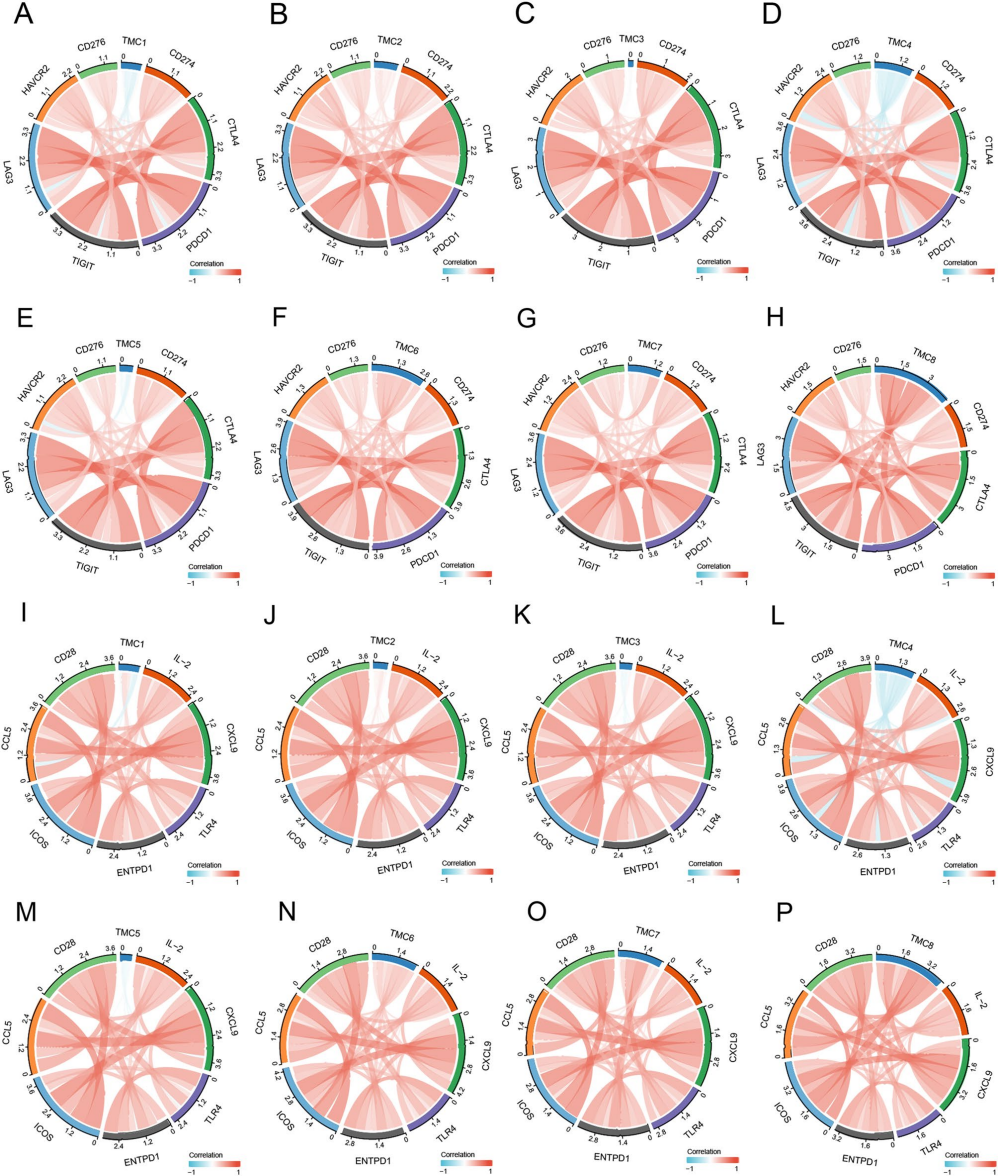
Relationship between TMC family expression and immune checkpoint genes in RCCC. (**A**–**P**) Associations between TMC family expression and PDCD1, CD274, CD276, CTLA-4, TIGIT, LAG3, HAVCR2, IL-2, CLCL9, TLR4, ENTPD1, ICOS, CCL5, and CD28 in RCCC.

We also conducted the co-expression analysis to explore the association between TMC family expression and immune checkpoint genes in RCCC using the R package “circlize” and TIMER2.0 web. In RCCC patients, the levels of TMC2, TMC6, TMC7, and TMC8 were positively correlated with PDCD1, CD274, CD276, CTLA-4, TIGIT, LAG3, HAVCR2, IL-2, CLCL9, TLR4, ENTPD1, ICOS, CCL5, and CD28, whereas the association between TMC4 and these immune checkpoints was negative (Fig. 7, Figures S4 and S5).

**Figure 8:**
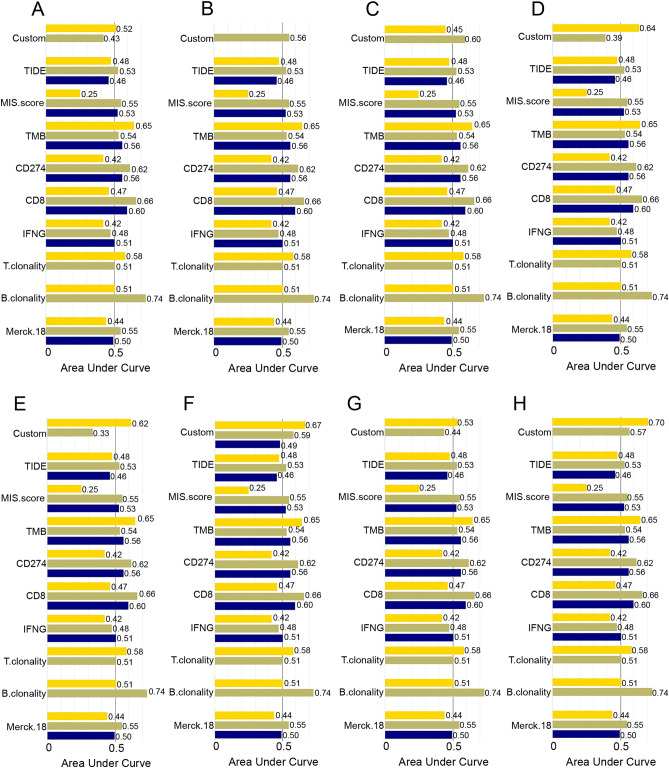
The biomarker relevance of TMCs. Bar plot showing the biomarker relevance of TMCs compared to standardized cancer immune evasion biomarkers in immune checkpoint blockade sub-cohorts (Yellow: Miao2018_ICB_Kidney_Clear; Brown: McDermott2018_PDL1_Kidney_Clear; Blue: Braun2020_PD1_Kidney_Clear). The area under curve was applied to evaluate the predictive performances of the test biomarkers on the ICB response status. (**A**–**H**) represents TMC family genes TMC1-8.

Then, we evaluated the biomarker relevance of TMCs by comparing them with standardized biomarkers based on their predictive power of response outcomes and OS of ICB sub-cohorts in RCCC patients. In Fig. 8, the AUC of TMC6 and TMC8 was greater than 0.5 in 2 of 3 RCCC ICB sub-cohorts (“Custom”). This result implied that TMC6 and TMC8 were more vital as predictive immunological biomarkers than other TMCs.

### Correlation analysis among TMC family members

**Figure 9:**
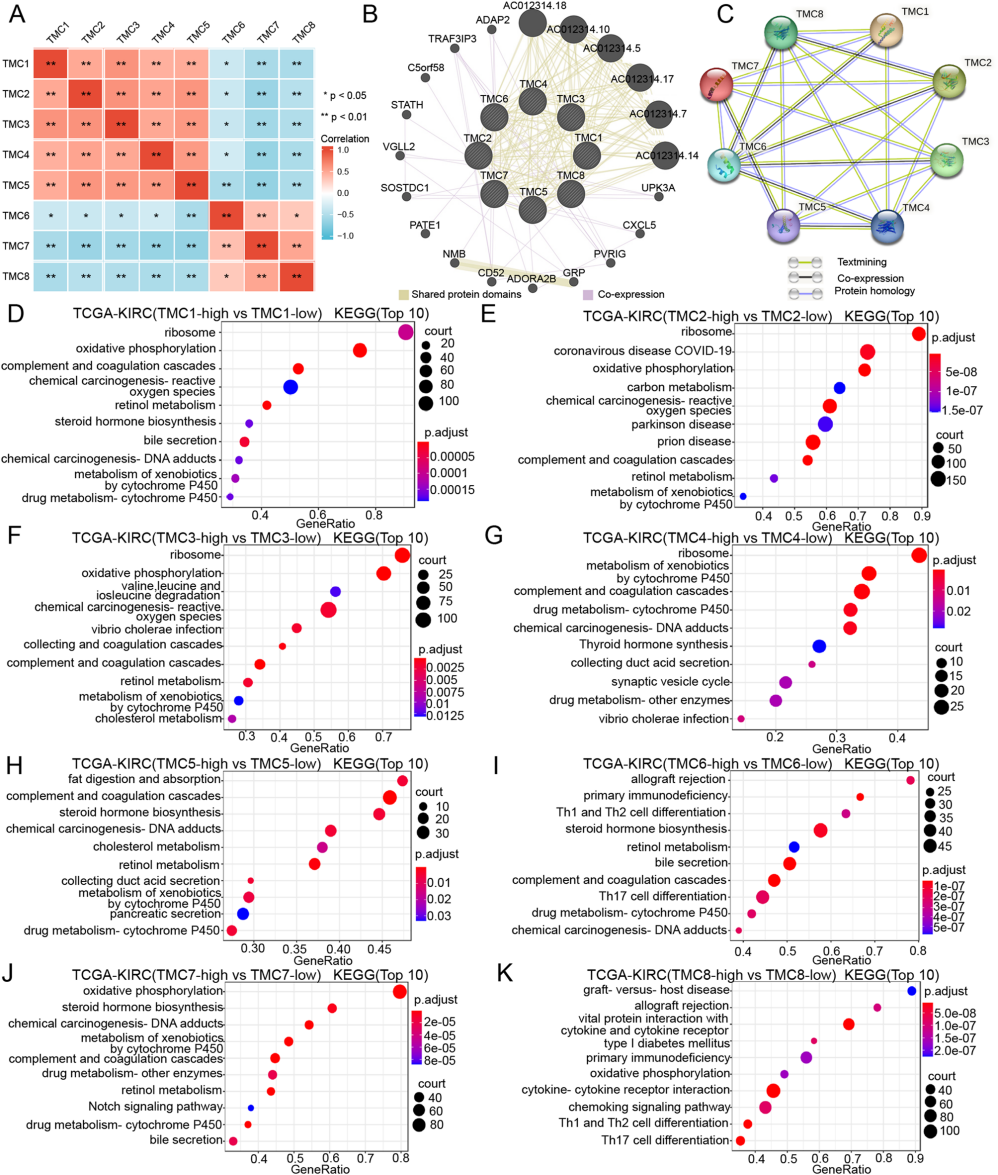
The Protein–protein interaction analysis and pathway analysis of TMC genes. (**A**) Co-expression analysis among TMC family members in RCCC. (**B**) The PPI analysis of TMCs (GeneMANIA). (**C**) The PPI analysis of TMCs (STRING 11.5). (**D**–**K**) The KEGG pathway analysis of TMCs based on TCGA-KIRC cohort.

A gene family is a group of genes with significant similarities encoding similar protein products, usually grouped by functional sets. In this part, we explored the relationship among TMC family members using co-expression analysis. The mRNA expression of TMC1, TMC2, TMC3, TMC4, and TMC5 was positively correlated with each other, as were the level of TMC6, TMC7, and TMC8. However, the mRNA levels of TMC1-5 and TMC6-8 were negatively correlated (Fig. 9A). Then the GeneMANIA website was then utilized to display the correlation between TMCs and explore potential interaction partners. In Fig. 9B, the expression of TMCs was closely correlated with UPK3A, CXCL5, PVRIG, GRP, ADORA2B, and so on. We next depicted the relationship between TMC genes using the STRING 11.5 database, as shown in Fig. 9C.

### Functional and pathway enrichment analysis of the TMC family in RCCC

Finally, we utilized the CAMOIP web to analyze the differences in cancer-related function and pathway between the TMCs-high and TMCs-low groups by the GSEA method and to predict TMCs-related phenotypes and signaling pathways.

Interestingly, “ribosome” ranked first in the Kyoto Encyclopedia of Genes and Genomes (KEGG) enrichment analysis of TMC1, TMC2, TMC3 and TMC4. Except for TMC8, the other TMCs were all involved in “complement and coagulation cascades.” Except for TMC4 and TMC8, the other TMCs were all engaged in “retinol metabolism.” It was worth noting that the top ten KEGG analysis results of TMC6 included “allograft rejection,” “primary immunodeficiency,” “Th1 and Th2 cell differentiation”, “complement and coagulation cascades” and “Th17 cell differentiation”. And the top ten KEGG enrichment analysis results of TMC8 included “graft-versus-host disease,” “allograft rejection,” “viral protein interaction with cytokine and cytokine receptor,” “primary immunodeficiency,” “cytokine-cytokine receptor interaction,” “chemokine signaling pathway,” “Th1 and Th2 cell differentiation” and “Th17 cell differentiation” (Fig. 9D,E,F,G,H,I,J,K**)**. Those results implied that TMC6 and TMC8 were closely related to immune response.

Gene Ontology (GO) biological process analysis revealed that almost all TMCs except TMC4 were involved in the immune response (Figure S6). GO cellular component analysis showed that most TMCs were involved in the composition of “immunoglobulin complexes,” apart from TMC2 and TMC4. Besides, TMC6 and TMC8 were also related to “T cell receptor complexes”(Figure S7). GO molecular function analysis demonstrated that TMC3, TMC5, TMC6, TMC7, and TMC8 were associated with “antigen-binding” and “immunoglobulin receptor binding” (Figure S8).

According to the results of KEGG and GO analysis, TMC6 and TMC8 shared many similarities, which is consistent with other previous articles. In a word, the all results concordantly showed the inseparable relationship between the TMC family and immune infiltration in RCCC.

## Discussion

With the rapid development of medicine, bioinformatics, as a new method to promote medical progress, can be based on extensive sample sequencing data for quick analysis of tumor-related genes, early diagnosis of tumors, prediction of clinical prognosis, and treatment guidance^33–36^. Several methods have been utilized to manage RCCC, such as surgery, radiotherapy, chemotherapy, targeted therapy, and immunotherapy^37^. The cure for RCCC depends on complete surgical resection of the tumor (radical nephrectomy). Radiotherapy and chemotherapy can only play an auxiliary role due to their inefficiency^10,11^. Although radical nephrectomy has been successfully used to treat RCCC, distant metastasis still occurs in 30% of patients after surgery, which is associated with high mortality^6^. Immune checkpoint inhibitors combined with tyrosine kinase inhibitors have become the first-line treatment for RCCC. However, only a subset of patients has a favorable response to treatment, with objective response rates varying from 41 to 71% among different combinations^38–40^. Therefore, it is necessary to explore new biomarkers as prognostic indicators or therapeutic targets for RCCC.

In the present study, by bioinformatics methods, we systematically analyze the expression level of TMC genes in normal human kidney and RCCC tissues, the relationship with patients 'prognosis, the correlation with clinical stage and histologic grade, the relevance with immune infiltrating cells and immunotherapy response, the involved signaling pathways, and the gene functions.

So far, TMC1 and TMC2 have been well studied because of their indispensable roles in hearing conduction. Growing evidence showed that TMC family may play an important role in cancer and may have great clinical values. Wen et al. found that the expression level of TMC3 negatively correlated with the recurrence risk of papillary thyroid carcinoma^41^. Our study showed that TMC3 expression is down-regulated and positively correlated with the immune infiltration of RCCC. At the same time, the expression of TMC3 negatively correlated with the OS of RCCC patients. TMC4 can be used as a prognostic marker for breast cancer, and its high expression was associated with a better prognosis^42^. In this study, TMC4 was lowly expressed in RCCC tissues. However, it did not have a significant connection with the prognosis and immune infiltration of RCCC patients. Overexpression of TMC5 was observed in intrahepatic cholangiocarcinoma^43^, prostate cancer^28^, hepatocellular carcinoma^44^, lung adenocarcinoma^45^ and chromophobe renal cell carcinoma^46^. But we found that the expression of TMC5 declined in RCCC tissues and negatively correlated with the survival and prognosis of RCCC patients.

In recent years, immune infiltrating cells in TME have been paid more attention for their crucial role in tumor genesis and development^47^. Tumor-associated immune cells are classified into two groups based on their function in tumors: tumor- antagonistic immune cells (such as CD8 + T cells, CD4 + T cells, NK cells, DCs, M1-type macrophages, and N1-polarized neutrophils) and tumor- promoting immune cells (such as regulatory T cells and myeloid suppressor cells)^48^. Mutations of TMC6 and TMC8 were not only reported to contribute to an increased risk of squamous cell skin cancer^30^ but also correlated to cervical cancer susceptibility^49^. Besides, TMC8 was reported as a prognostic biomarker for head and neck squamous cancer^50^. In this study, TMC6 and TMC8 showed a lot of similar properties and were positively correlated with the degree of immune infiltration in RCCC. Our results were consistent with a previous study, which found that TMC6 and TMC8 positively associated with macrophages, B cells, and T cells in most cancer types^32^, suggesting that TMC genes may participate in the immunity of RCCC.

Tumor-antagonistic immune cells tend to kill tumor cells in the early stages of tumorigenesis. Still, tumor cells eventually escape immune surveillance through various mechanisms and even inhibit the cytotoxic effects of immune cells^51^. One mechanism is that tumor cells express immune checkpoint genes, such as PD1 expressed on T cells, which binds to PD-L1 ligands on the surface of tumor cells, causing T cells to silence and fail to kill the tumor^52^. TMC7 was identified as a potential prognostic biomarker for pancreatic cancer, and its high expression was associated with a poor prognosis^31^. An early study said TMC7 expression negatively correlated with CTLA4, HAVCR2, LAG3, PDCD1, and TIGIT expression in head and neck squamous cell carcinoma^53^. In contrast, our research came to the opposite conclusion in RCCC. The levels of TMC2, TMC6, TMC7, and TMC8 positively correlated with PDCD1, CD274, CD276, CTLA-4, TIGIT, LAG3, HAVCR2, IL-2, CLCL9, TLR4, ENTPD1, ICOS, CCL5, and CD28. In addition, the level of TMC7 was higher in RCCC samples, demonstrating a better prognosis. This finding suggests that the TMC family not only plays a role in the immune cell infiltration of RCCC but also has a certain correlation with the expression of numerous immunological checkpoints. In a word, the TMC family not only predicts tumor immune cell infiltration levels but also serves as a predictor of immune checkpoint expression, which may play a role in the therapeutic efficacy prediction of immunotherapy. However, all of these findings are estimated by bioinformatics and require further experimental validation.

The present study had certain limitations. The association between the expression of some TMCs and OS in RCCC patients was inconsistent. The correlation strength between TMCs and the infiltration degree of some immune cells in RCCC was only weak to moderate. The altered states of TMCs, including mutations and methylation, are also associated with cancer prognosis and immune infiltration. Further exploration of the molecular mechanisms by which TMCs affect RCCC development is also needed. Besides, our current study just provided insufficient evidence that the TMC family may be regarded as therapeutic targets for RCCC. In the future, we will perform relevant and targeted therapeutic drug screening based on different characteristics of TMC family members via the connectivity map (CMAP). The database studies the chemical structure of drugs through the ratio of gene expression data^54^.

In summary, this was the first and most comprehensive investigation of the expression profiles and clinical significance of TMC family members in patients with RCCC. The present study reported that variations in the expression of TMCs correlated with RCCC patients’ survival, which suggested that the level of TMCs could predict tumor prognosis. Furthermore, this research demonstrated that the extent of immune cell infiltration and the expression level of immune checkpoint genes correlated with the expression of TMCs in RCCC. Therefore, these results provided insight into the potential function of the TMC genes in tumor immunology and their potential as predictive biomarkers for RCCC.

## Conclusion

Our study showed the prognosis values of the TMC family in RCCC. Thus, we may regard the TMC family members as novel biomarkers to predict potential prognosis and immunotherapeutic response in RCCC patients, paving the way for further investigation of the tumor-infiltrating mechanisms and therapeutic potentials of TMCs in RCCC.

## Materials and methods

### Data collection

We used the UCSC Xena (https://xenabrowser.net/) to download TCGA-KIRC and GTEx-Kidney data, including RNAseq date, survival data, and clinical data.

### Gene expression analysis

RNAseq data from TCGA and GTEx databases were transformed to Transcripts per million (TPM) and then showed with log_2_(TPM + 1) by using the R package “ggplot2”. Then the Wilcox test was used to evaluate the differences in mRNA levels of the TMC family between 100 normal tissues and 531 RCCC tissues. The mRNA expression of TMCs in 72 pairs of RCCC and adjacent samples was evaluated by paired sample t-test. The University of Alabama at Birmingham Cancer data analysis Portal (UALCAN, http://ualcan.path.uab.edu/index.html) and Gene Expression Profiling Interactive Analysis version 2.0 (GEPIA2, http://gepia2.cancer-pku.cn/) were used to display mRNA expression levels of TMC family members between RCCC and healthy renal tissues.

### Survival analysis, clinical correlation analysis, and immune infiltration analysis

The overall survival (OS), disease-specific survival (DSS), and progress free interval (PFI) curves were displayed by using package “survminer” and “survival.” The Kaplan–Meier (KM, http://kmplot.com) curves were used to analyze the correlation between TMC family expression and OS in RCCC patients. The expression of TMCs in various pathological stages of RCCC was obtained using the “Pathological Stage Plot” module in GEPIA2. Receiver operating characteristic (ROC) curves of TMCs were developed using the package “pROC.” The packages “immuneeconv” and “ggplot2” were used to assess the reliable results of immune score evaluation. The package “circlize” and Tumor Immune Estimation Resource 2.0 (TIMER2, http://timer.cistrome.org/) were utilized to plot spearman’s correlation coefficient between TMC family expression and immune checkpoint genes. The package “ESTIMATE” was used to evaluate the stromal and immune scores in RCCC. The predictive power of TMCs in immunotherapy response was compared with some other standardized biomarkers of tumor immune response, including T-cell clonality (T. Clonality), B-cell clonality (B. Clonality), Tumor Immune Dysfunction and Exclusion (TIDE), estimating the microsatellite instability (MSI) score, tumor mutational burden (TMB), cluster of differentiation 8 (CD8), cluster of differentiation 274 (CD274), and interferon-γ (IFNG) using the biomarker evaluation module of the TIDE server (http://tide.dfci.harvard.edu/).

### Functional enrichment and protein–protein interaction(PPI) network analysis

The Comprehensive Analysis on Multi-Omics of Immunotherapy in Pan-cancer (CAMOIP, https://www.camoip.net/) was used to analyze the differences in processes and pathways between the TMCs-high and TMCs-low groups by the gene set enrichment analysis (GSEA) method and to predict TMCs-related phenotypes and signaling pathways. This study investigated the interaction between TMCs and protein using the Search Tool for Recurring Instances of Neighbouring Genes 11.5 (STRING 11.5, https://cn.string-db.org/) with the minimum required interaction score set as 0.15. GeneMANIA (http://www.genemania.org) is an online site which have abundant genetic information and can analyze the correlation among several genes. With the help of GeneMANIA website, we further displayed the interaction network among TMCs and explored the several potential interaction partners.

### Cell cines and cell culture

Human renal cortex proximal convoluted tubule epithelial cells (HK2) and RCCC cells (786–0 and ACHN) were provided by Cell Bank/Stem Cell Bank, Chinese Academy of Sciences. HK2 and ACHN were cultivated in DMEM high glucose media with 1% penicillin–streptomycin and 10% fetal bovine serum. 786-O was cultivated in RPMI-1640 media with 1% penicillin–streptomycin and 10% fetal bovine serum. All cells were cultured in a humidified incubator at 37 °C with 5% CO_2_.

### Real-time PCR Assay

Total cellular RNA was extracted with a cellular RNA extraction kit from Shandong Sparkjade Biotechnology Co., Ltd. (Jinan, China) and then reverse transcribed into cDNA by using the Evo M-MLV RT Kit from Accurate Biotechnology (Hunan) Co., Ltd. (Changsha, China). Hieff®qPCR SYBR Green Master Mix (11201ES08) from Yeasen Biotechnology (Shanghai) Co., Ltd. (Shanghai, China) was then used for real-time PCR. The real-time PCR was performed using a Bio-Rad CFX96 real-time PCR system (Bio-Rad, Hercules, CA, USA). The results were calculated by the comparative Ct method. All primers were designed by Primer Premier 6 and synthesized by Sangon Biotech (Shanghai). Primer sequences are shown in Table 2.

**Table 2:** Sequence of real-time PCR primers.

The name of the primer	Primer sequences
TMC1	F: TGAAATGGCTACTGGGACG
	R: GAGGCTGGGAGCAAAGAA
TMC2	F: GGTTAAGCGATCTCAGCAATT
	R: AGGGCAGCGATGGTTTC
TMC3	F: CTGGCGTTGCCTCCTATT
	R: CAGACGAAACCTGCTCCTT
TMC4	F: CCCTTGTCCAGGAGTTGC
	R: GGCGAGGCGAAGAAACA
TMC5	F: TCGCTGTCTTCTGTGCTGA
	R: CCATCGGTCCGTCTTAGTT
TMC6	F: GCATGGCTCACTCTTTCGG
	R: GCTTCTGCGTCACCTTGTAGTC
TMC7	F: GCCAACCGACTCTTACAGC
	R: TGAAAGGAATGAGCACGAA
TMC8	F: TGCTGGACATCGTGGCG
	R: AGGGTGCTGAGTAGTTGGTGAA
β-actin	F: CGTGCGTGACATTAAGGAGAAG
	R: GGAAGGAAGGCTGGAAGAGTG

### Statistical analysis

All data were displayed by mean plus or minus standard deviation. Statistical analysis was managed using Prism 9 and SPSS 13. The value of *p* < 0.05 was considered significant (*p < 0.05, **p < 0.01, ***p < 0.001).

## Supplementary Information

Supplementary Information.

## Data Availability

The data which support the findings of our study are all openly available from the UCSC Xena (https://xenabrowser.net/), Kaplan–Meier(http://kmplot.com), UALCAN (http://ualcan.path.uab.edu/index.html), GEPIA2(http://gepia2.cancer-pku.cn/), TIMER2.0 (http://timer.cistrome.org/), TIDE (http://tide.dfci.harvard.edu/), STRING (https://cn.string-db.org/), GeneMANIA (http://www.genemania.org) and CAMOIP (https://www.camoip.net/) database.
