# Supplementary material for: Comprehensive analysis of the prognosis and immune infiltration of TMC family members in renal clear cell carcinoma
Source: Sci Rep. 2023 Jul 19;13:11668. doi: 10.1038/s41598-023-38914-z (PMC10356759; doi:10.1038/s41598-023-38914-z)
Supplement: Supplementary file 1 — Supplementary Information. [file 41598_2023_38914_MOESM1_ESM.zip › supplementary material/supplementary material.docx]

**Comprehensive analysis of the prognosis and immune infiltration of TMC family members in renal clear cell carcinoma****Authors:** Wenbin Tang^1,#^, Zhiyuan Shi^2,#^, Yasheng Zhu^1,#^, Zhengda Shan^4^, Aimin Jiang^3^, Anbang Wang^1^, Ming Chen^1^, Yi Bao^1^, Guanqun Ju^1^, Weidong Xu^1,*^ and Junkai Wang^1,*^

**Affiliations:** 1 Department of Urology, Changzheng Hospital, Naval Medical University, NO.415 Fengyang Road, Shanghai 200003, China2 Department of Urology, Xiang’an Hospital of Xiamen University, School of Medicine, Xiamen University, NO.4221 Xiang’an South Road, Xiamen 361101, Fujian Province, China3 Department of Urology, Changhai Hospital, Naval Medical University, NO.168 Changhai Road, Shanghai 200082, China4 School of Medicine, Sun Yat-Sen University, NO.66 Gongchang Road, Shenzhen 518107, Guangdong Province, China# These authors contributed equally to this work.*** Corresponding authors:**Prof. Weidong Xu, Department of Urology, Changzheng Hospital, Naval Medical University, NO.415 Fengyang Road, Shanghai 200003, China; E-mail: [shhxwd@163.com](mailto:shhxwd@163.com)

Prof. Junkai Wang, Department of Urology, Changzheng Hospital, Naval Medical University, NO.415 Fengyang Road, Shanghai 200003, China; E-mail: [onealstorm@smmu.edu.cn](mailto:onealstorm@smmu.edu.cn)

**Supplementary Figures**


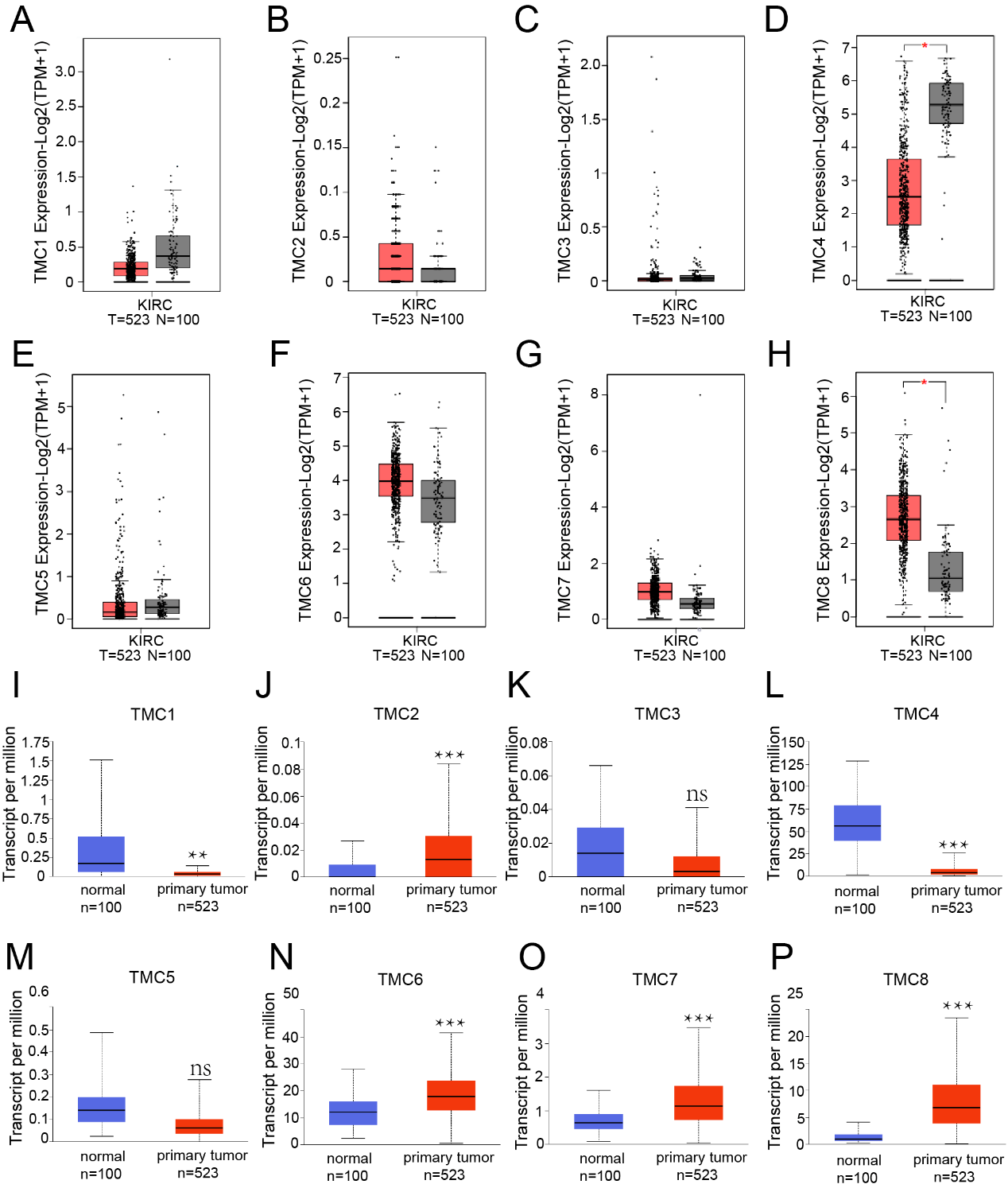

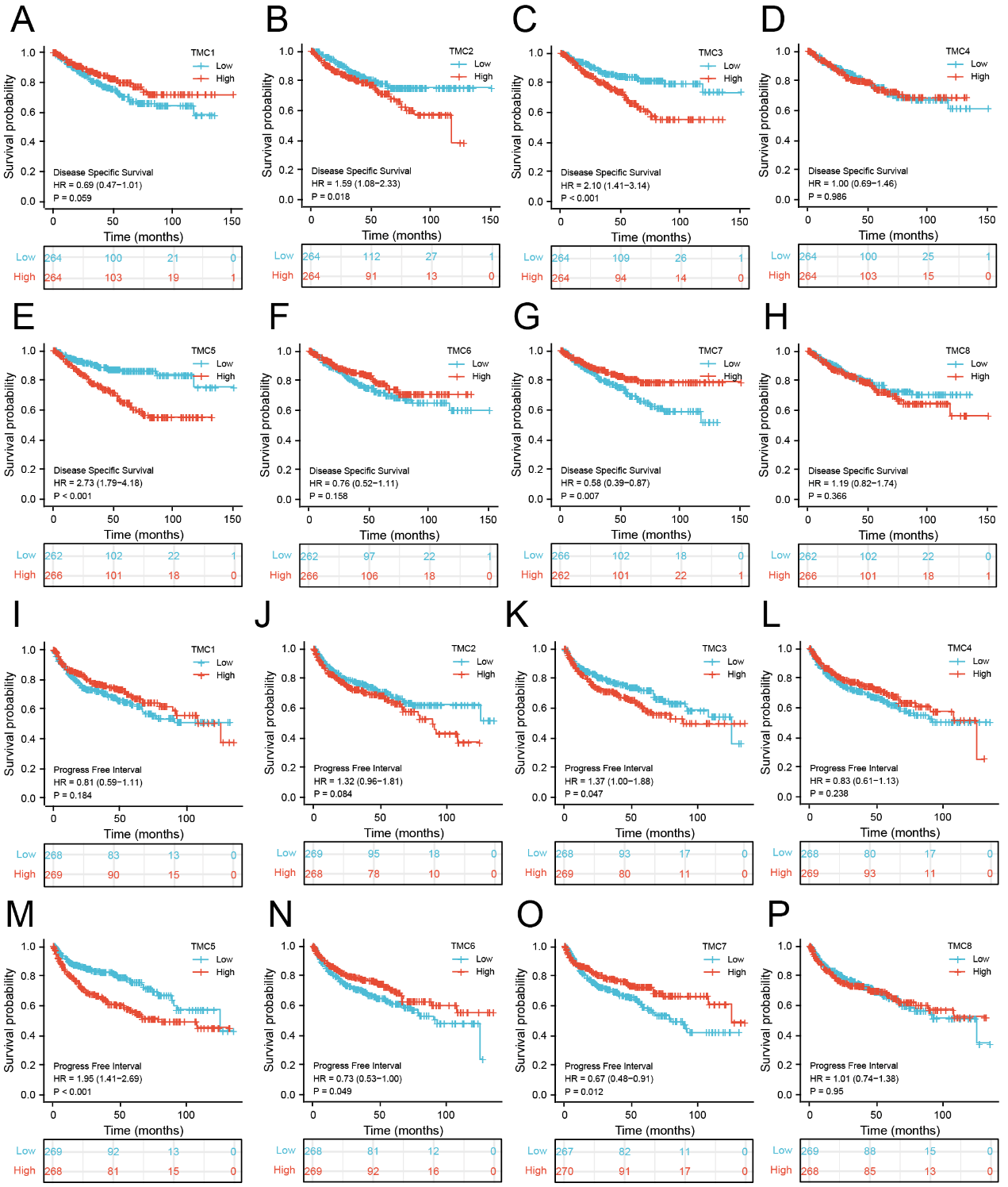

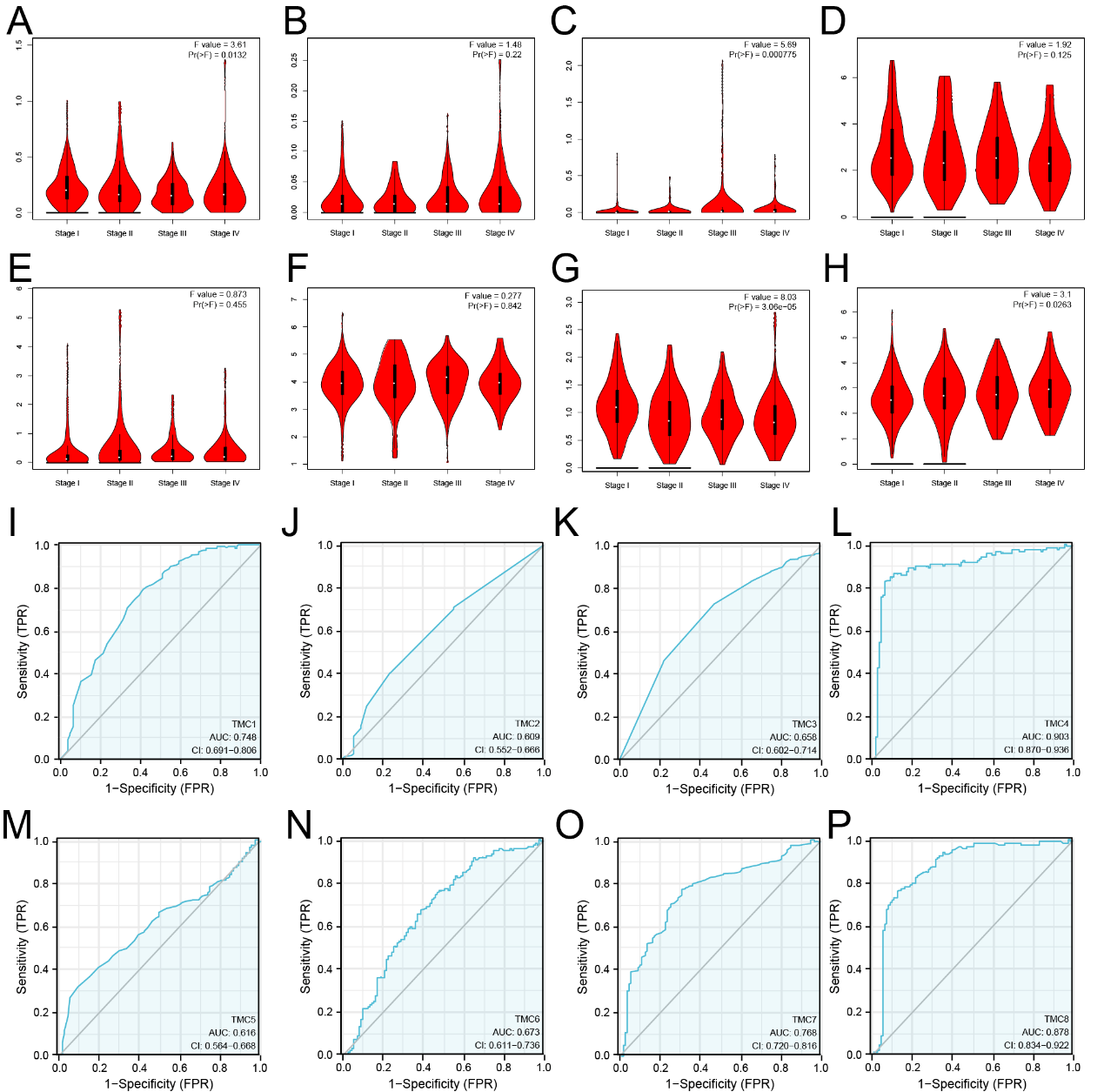

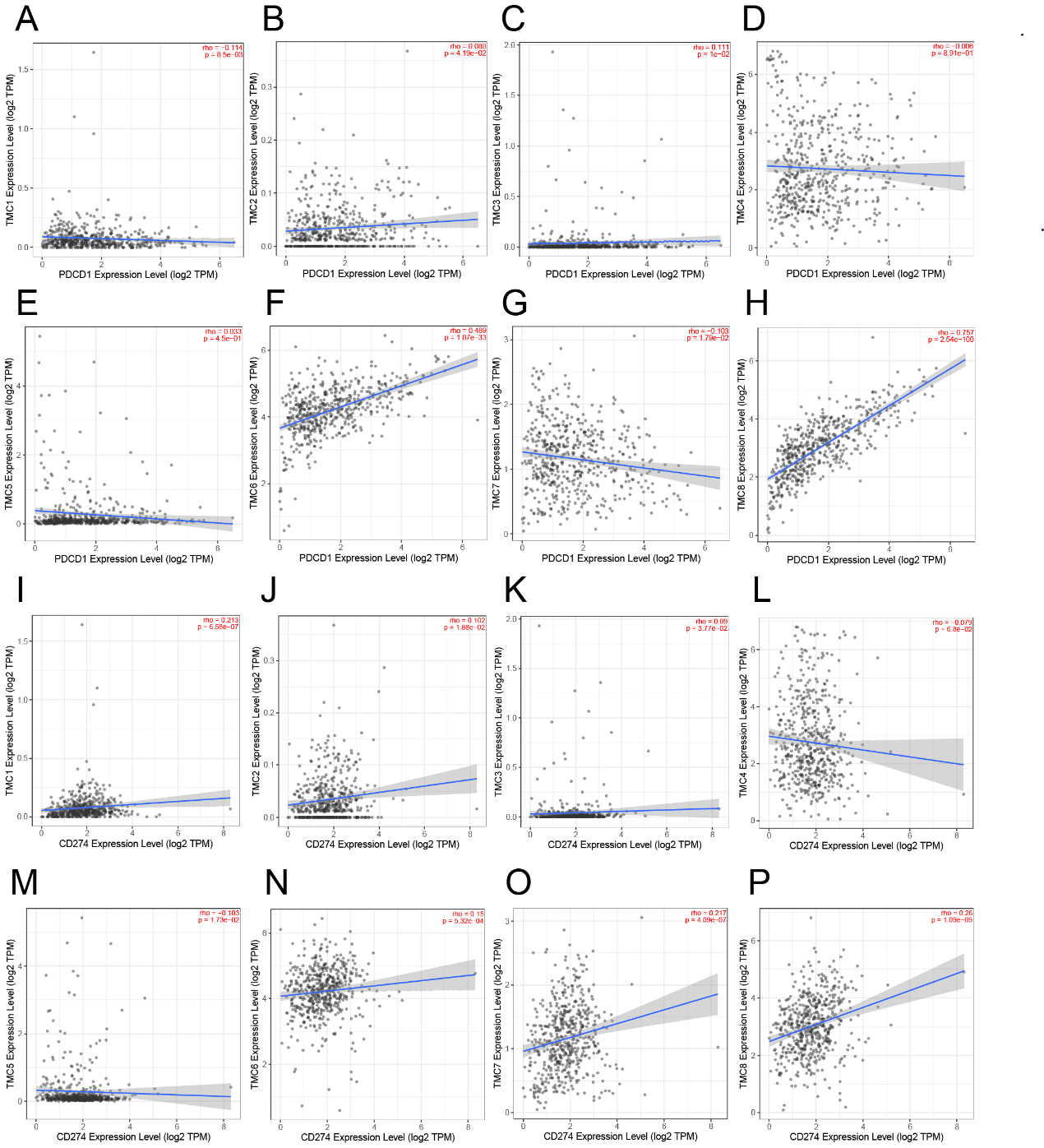

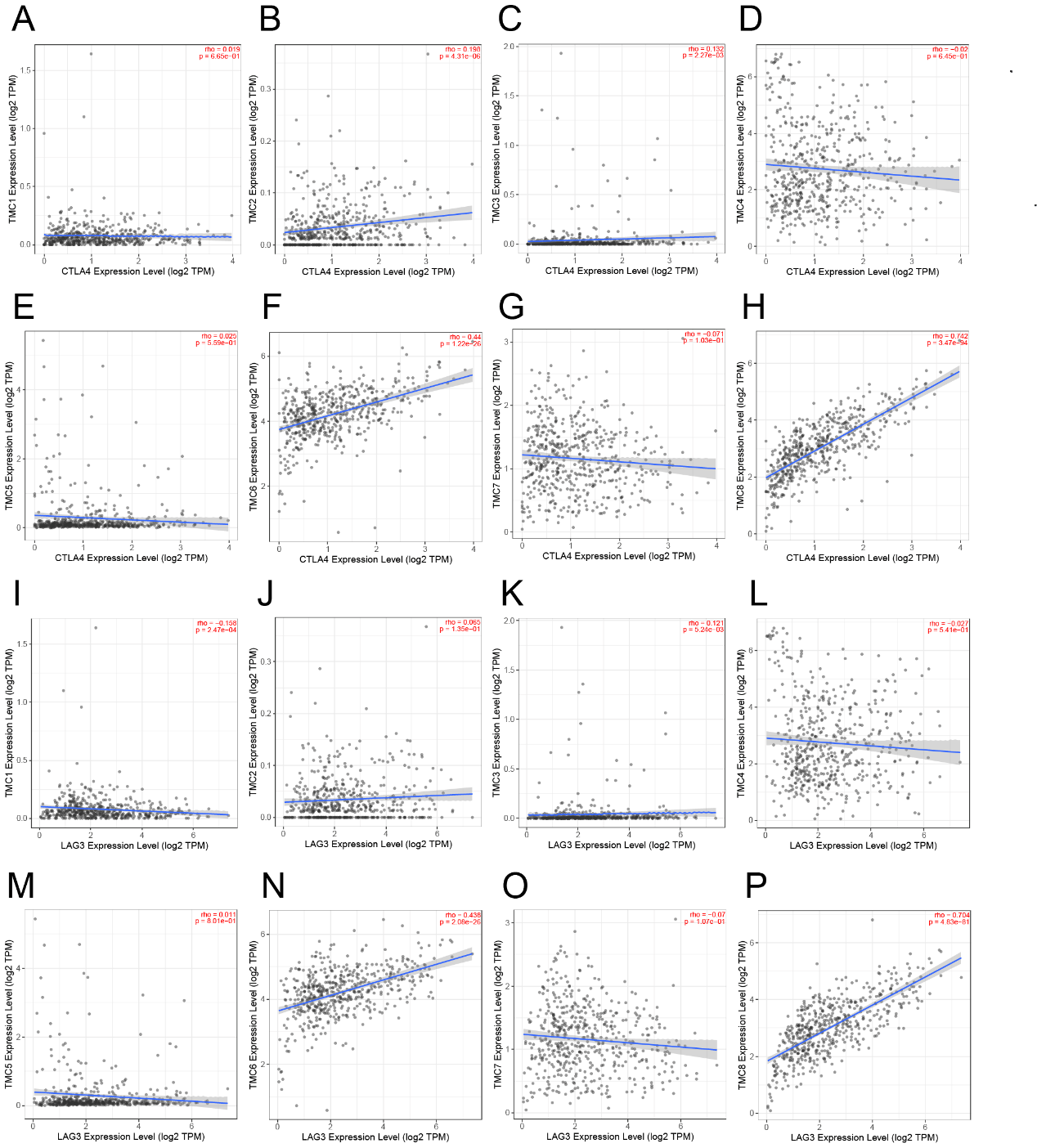

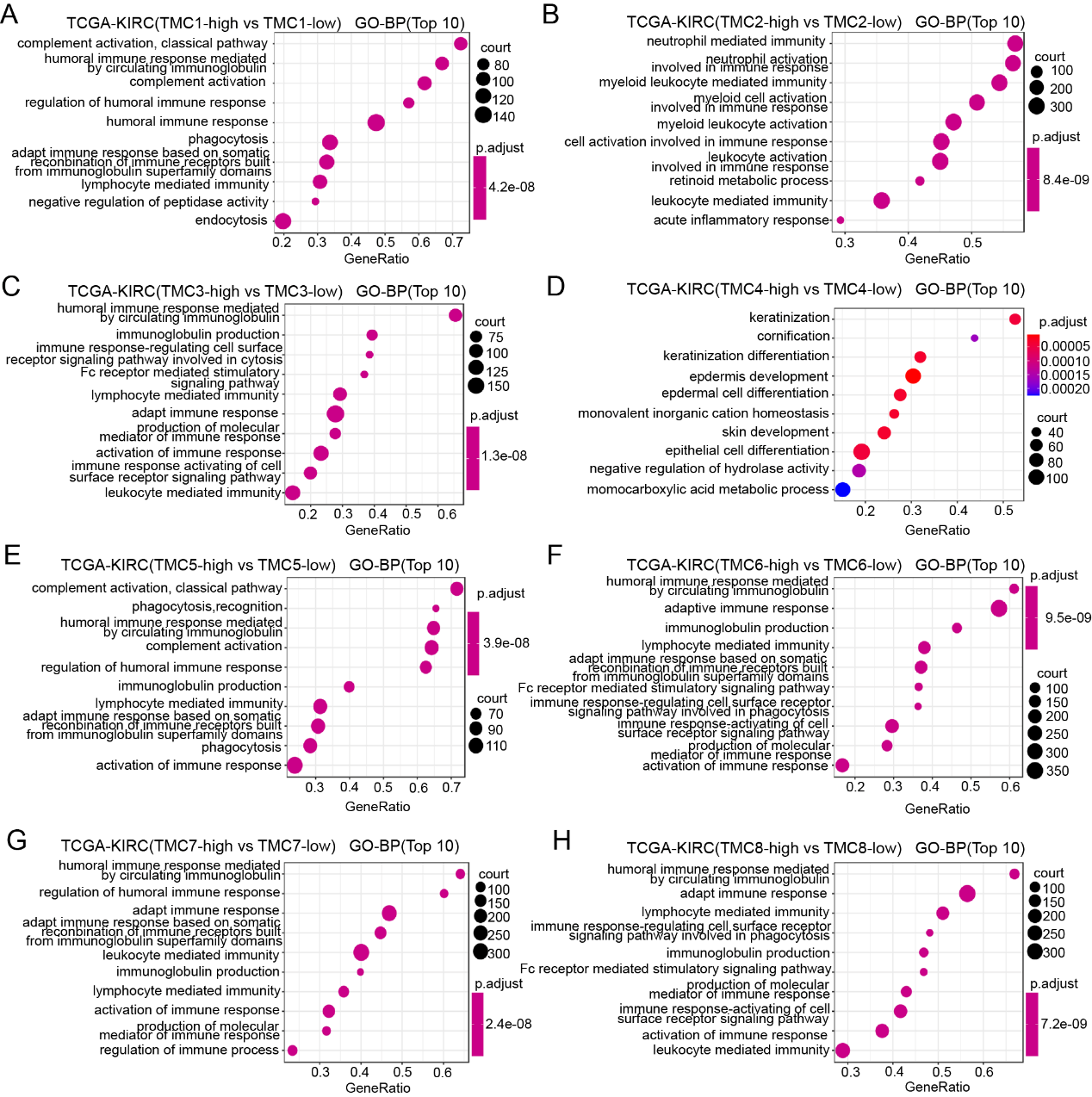

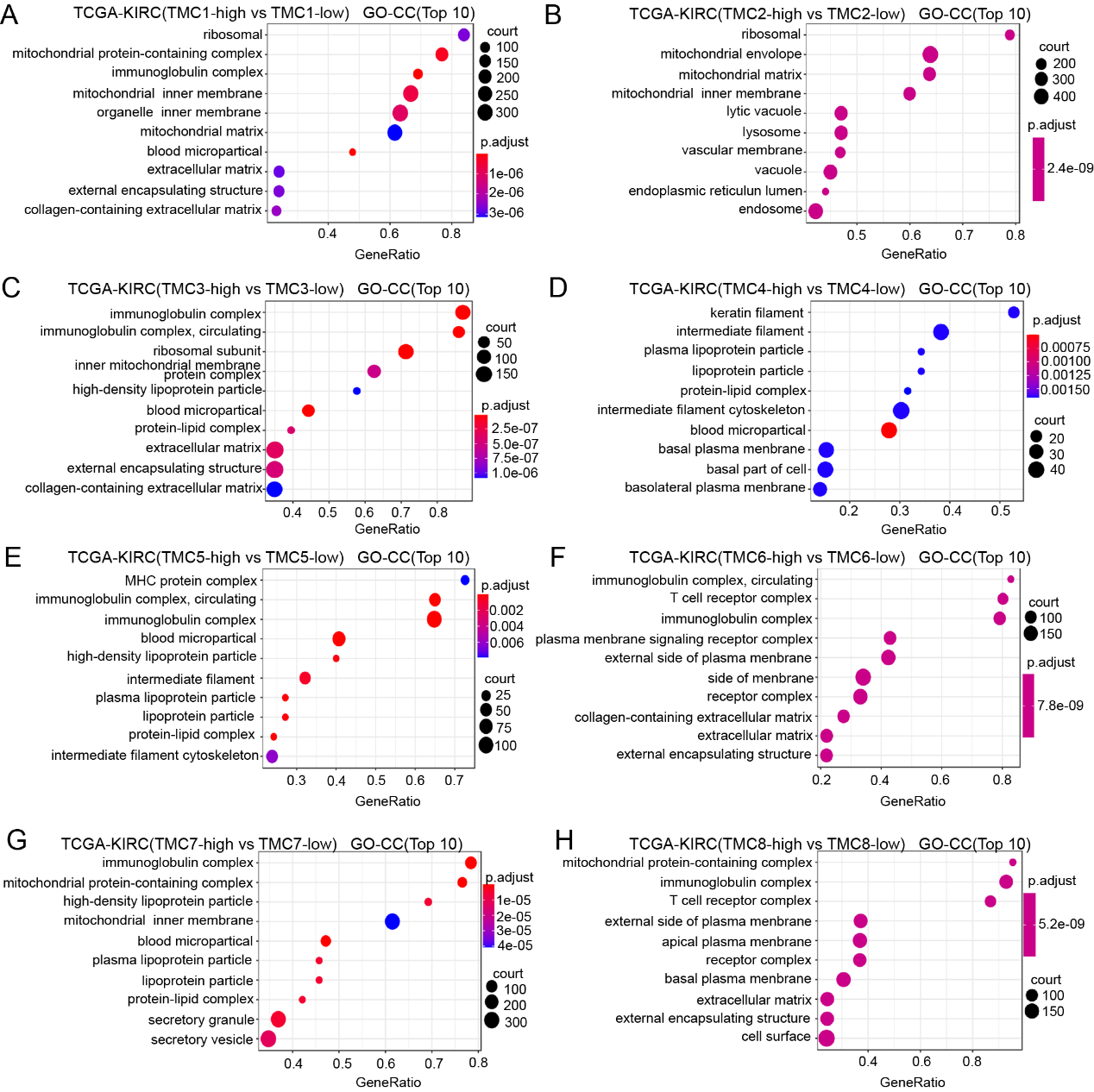

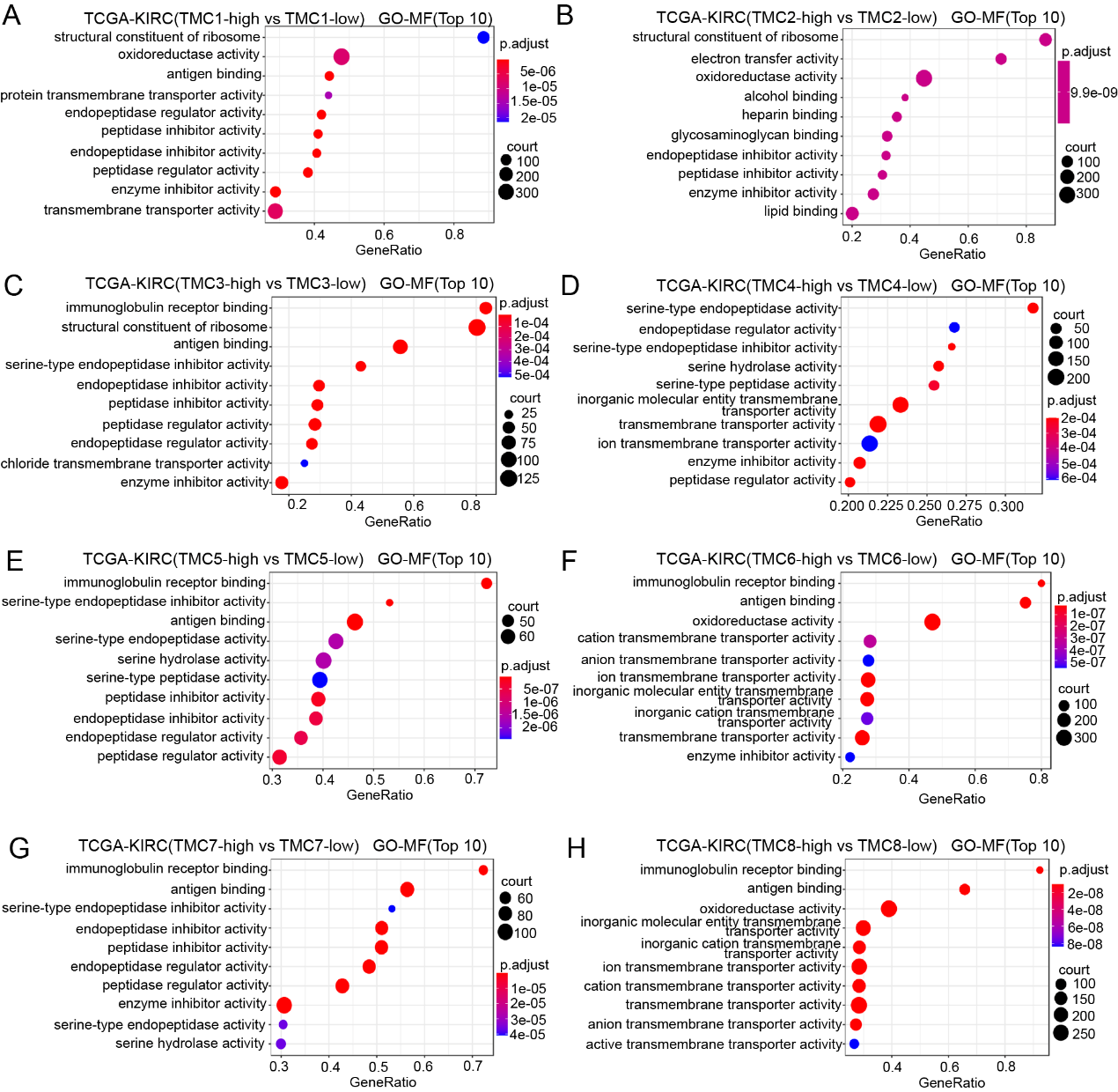


**Supplementary Figure legends**

**Figure S1**:

The mRNA level of TMCs in RCCC tissues. *p < 0.05, **p < 0.01, ***p < 0.001, ns: no statistically significant. (A-H) The mRNA expression of TMCs in RCCC from the GEPIA database. Tumor tissues are shown in red, and normal tissues are shown in gray. (I-P) The mRNA expression of TMCs in RCCC from the UALCAN database.

**Figure S2**:

Effect of TMC family expression on survival of RCCC patients. (A-H) Association between TMC family expression and RFS of RCCC patients. (I-P) Association between TMC family expression and PFI of RCCC patients.

**Figure S3**:

Correlation analysis between TMC family expression and clinical stage of RCCC patients. (A–K) represents TMC genes TMC1–8. The graphs were generated using the GEPIA. (I-P) ROC curve of TMCs showing their diagnostic capability for RCCC.

**Figure S4**:

Correlation of TMC family expression and the level of PDCD1 and CD274. (A-H) Associations between TMC expression and the level of PDCD1. (I-P) Associations between TMC expression and the level of CD274. The scatter plots were generated using the TIMER 2.0 database.

**Figure S5**:

Correlation of TMC family expression and the level of CTLA4 and LAG3. (A-H) Associations between TMC expression and the level of CTLA4. (I-P) Associations between TMC expression and the level of LAG3. The scatter plots were generated using the TIMER 2.0 database.

**Figure S6:** The GO-BP functional enrichment analysis of the TMCs based on TCGA-KIRC cohort. (A-H) represents TMC genes TMC1-8.**Figure S7:** The GO-CC functional enrichment analysis of the TMCs based on TCGA-KIRC cohort. (A-H) represents TMC genes TMC1-8.**Figure S8:** The GO-MF functional enrichment analysis of the TMCs based on TCGA-KIRC cohort. (A-H) represents TMC genes TMC1-8.
