# Supplementary material for: Comprehensive analysis of the prognosis and immune infiltration of TMC family members in renal clear cell carcinoma
Source: Sci Rep. 2023 Jul 19;13:11668. doi: 10.1038/s41598-023-38914-z (PMC10356759; doi:10.1038/s41598-023-38914-z)
Supplement: Supplementary file 1 — Supplementary Information. [file 41598_2023_38914_MOESM1_ESM.zip › supplementary material/supplementary material.pdf]

Comprehensive analysis of the prognosis and immune infiltration of TMC family members in renal clear cell carcinoma

Authors:

Wenbin Tang[1,#], Zhiyuan Shi[2,#], Yasheng Zhu[1,#], Zhengda Shan[4], Aimin Jiang[3], Anbang Wang[1], Ming Chen[1], Yi Bao[1], Guanqun Ju[1], Weidong Xu[1,*] and Junkai Wang[1,*]

Affiliations:

1 Department of Urology, Changzheng Hospital, Naval Medical University, NO.415 Fengyang Road, Shanghai 200003, China

2 Department of Urology, Xiang'an Hospital of Xiamen University, School of Medicine, Xiamen University, NO.4221 Xiang'an South Road, Xiamen 361101, Fujian Province, China

3 Department of Urology, Changhai Hospital, Naval Medical University, NO.168 Changhai Road, Shanghai 200082, China

4 School of Medicine, Sun Yat-Sen University, NO.66 Gongchang Road, Shenzhen 518107, Guangdong Province, China

# These authors contributed equally to this work.

*** Corresponding authors:**

Prof. Weidong Xu, Department of Urology, Changzheng Hospital, Naval Medical University, NO.415 Fengyang Road, Shanghai 200003, China; E-mail: shhxwd@163.com

Prof. Junkai Wang, Department of Urology, Changzheng Hospital, Naval Medical University, NO.415 Fengyang Road, Shanghai 200003, China; E-mail: onealstorm@smmu.edu.cn

Supplementary Figures

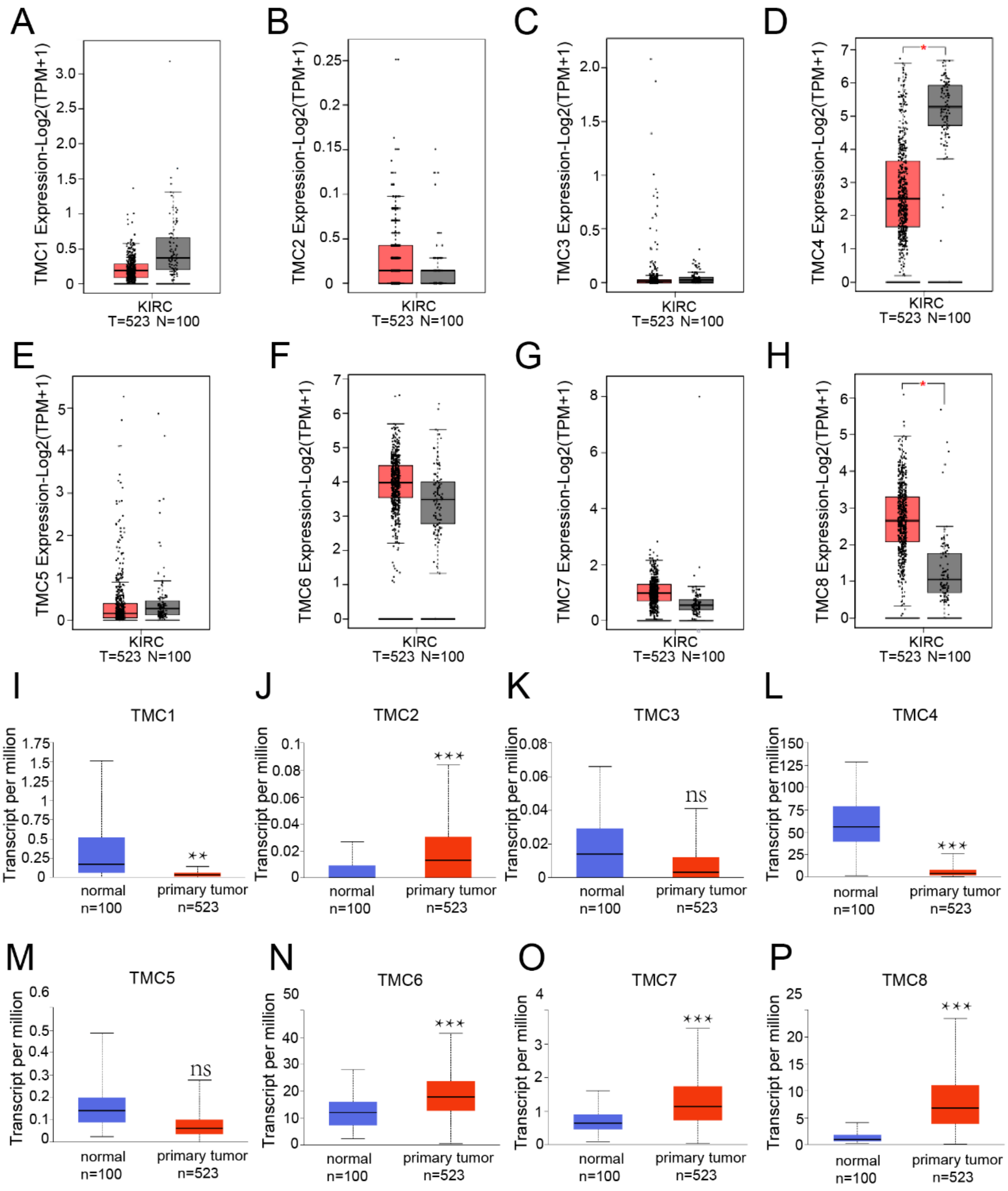

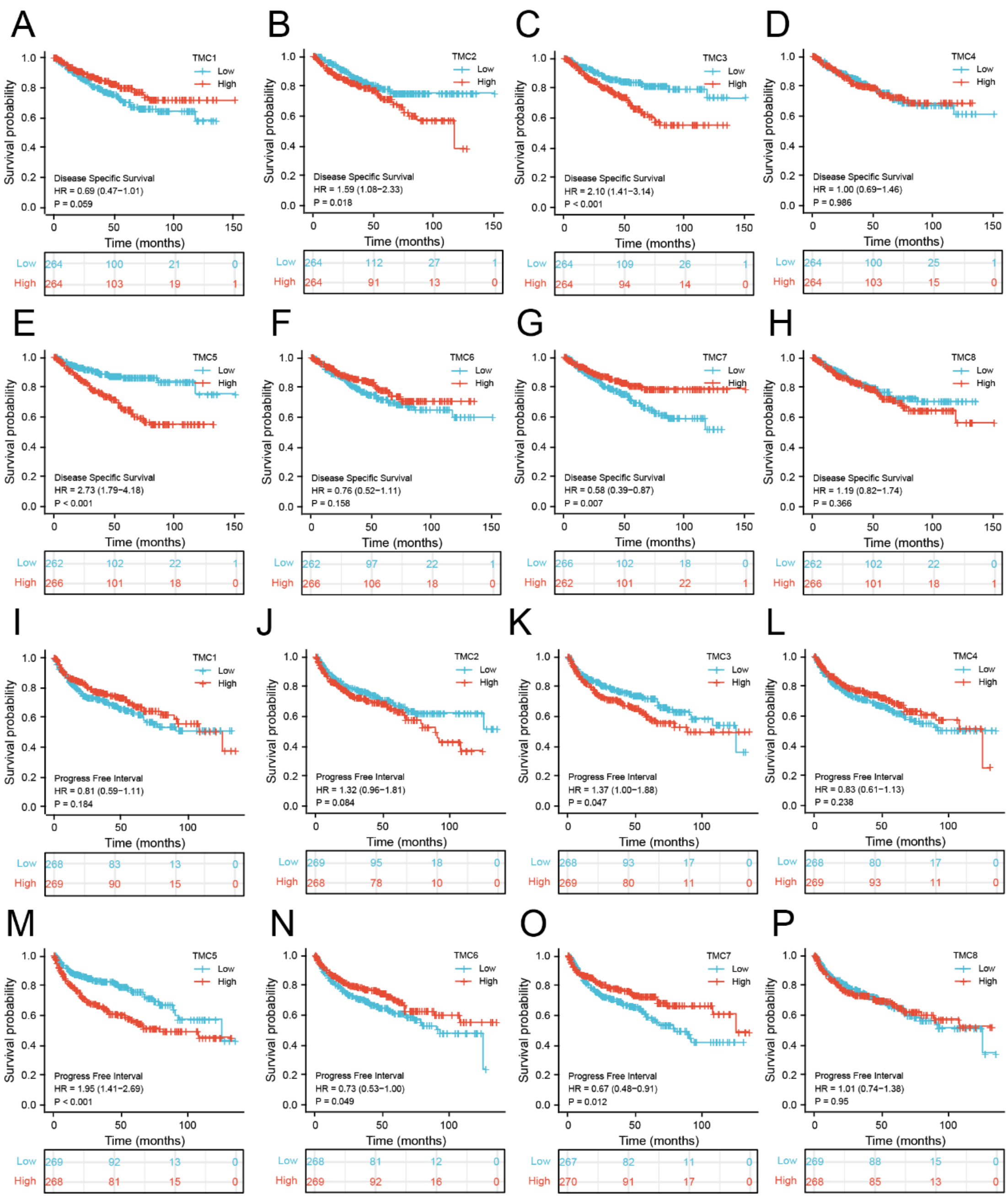
A
TMC1
Low
High
Disease Specific Survival
HR = 0.69 (0.47−1.01)
P = 0.059
Survival probability
Time (months)
Low 264 100 21 0
High 264 103 19 1
B
TMC2
Disease Specific Survival
HR = 1.59 (1.08−2.33)
P = 0.018
Low 264 112 27 1
High 264 91 13 0
C
TMC3
Disease Specific Survival
HR = 2.10 (1.41−3.14)
P < 0.001
Low 264 109 26 1
High 264 94 14 0
D
TMC4
Disease Specific Survival
HR = 1.00 (0.69−1.46)
P = 0.986
Low 264 100 25 1
High 264 103 15 0
E
TMC5
Disease Specific Survival
HR = 2.73 (1.79−4.18)
P < 0.001
Low 262 102 22 1
High 266 101 18 0
F
TMC6
Disease Specific Survival
HR = 0.76 (0.52−1.11)
P = 0.158
Low 262 97 22 1
High 266 106 18 0
G
TMC7
Disease Specific Survival
HR = 0.58 (0.39−0.87)
P = 0.007
Low 266 102 18 0
High 262 101 22 1
H
TMC8
Disease Specific Survival
HR = 1.19 (0.82−1.74)
P = 0.366
Low 262 102 22 0
High 266 101 18 1
I
TMC1
Progress Free Interval
HR = 0.81 (0.59−1.11)
P = 0.184
Low 268 83 13 0
High 269 90 15 0
J
TMC2
Progress Free Interval
HR = 1.32 (0.96−1.81)
P = 0.084
Low 269 95 18 0
High 268 78 10 0
K
TMC3
Progress Free Interval
HR = 1.37 (1.00−1.88)
P = 0.047
Low 268 93 17 0
High 269 80 11 0
L
TMC4
Progress Free Interval
HR = 0.83 (0.61−1.13)
P = 0.238
Low 268 80 17 0
High 269 93 11 0
M
TMC5
Progress Free Interval
HR = 1.95 (1.41−2.69)
P < 0.001
Low 269 92 13 0
High 268 81 15 0
N
TMC6
Progress Free Interval
HR = 0.73 (0.53−1.00)
P = 0.049
Low 268 81 12 0
High 269 92 16 0
O
TMC7
Progress Free Interval
HR = 0.67 (0.48−0.91)
P = 0.012
Low 267 82 11 0
High 270 91 17 0
P
TMC8
Progress Free Interval
HR = 1.01 (0.74−1.38)
P = 0.95
Low 269 88 15 0
High 268 85 13 0

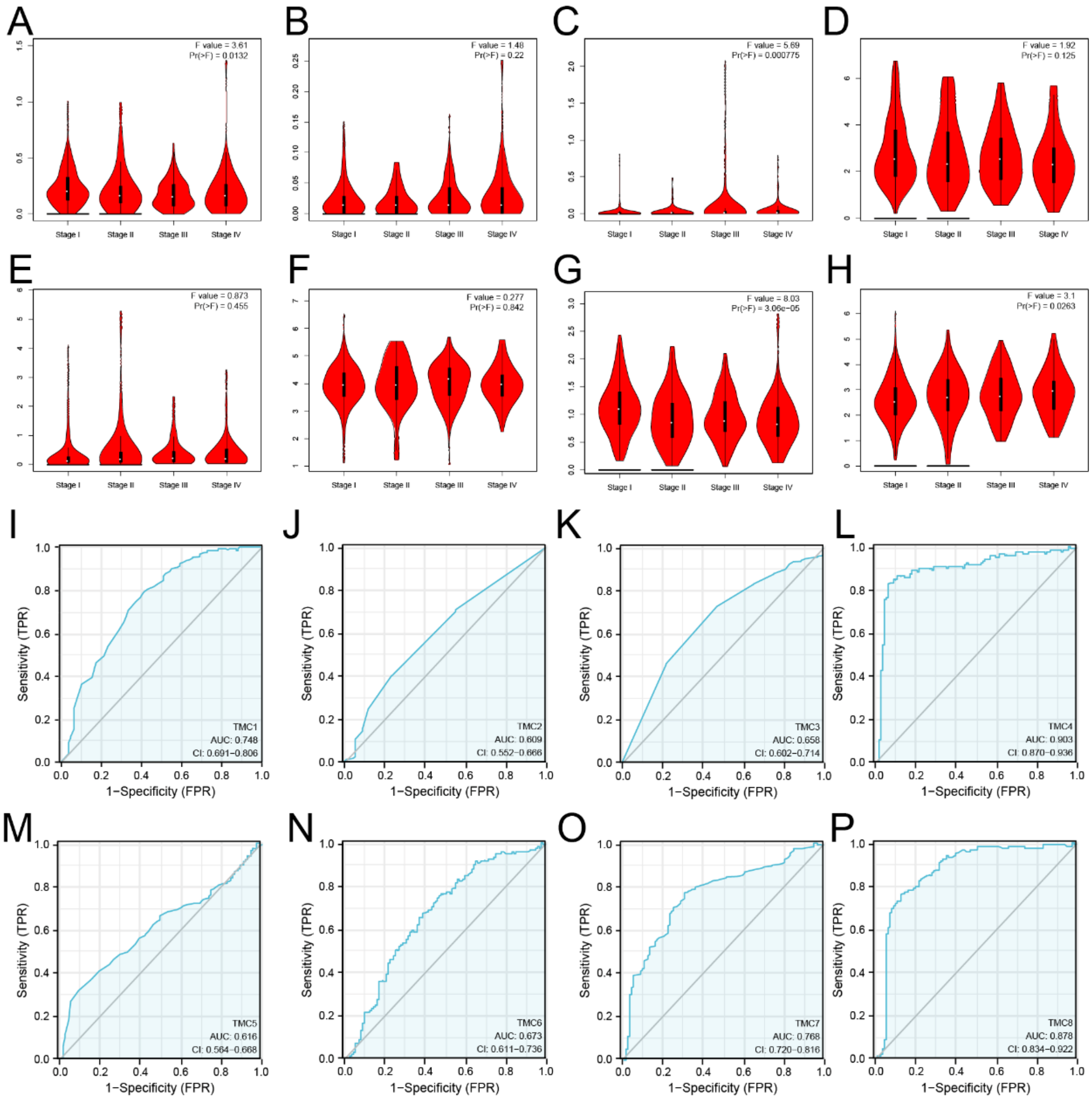
A
F value = 3.61
Pr(>F) = 0.0132
Stage I
Stage II
Stage III
Stage IV
B
F value = 1.48
Pr(>F) = 0.22
C
F value = 5.69
Pr(>F) = 0.000775
D
F value = 1.92
Pr(>F) = 0.125
E
F value = 0.873
Pr(>F) = 0.455
F
F value = 0.277
Pr(>F) = 0.842
G
F value = 8.03
Pr(>F) = 3.06e−05
H
F value = 3.1
Pr(>F) = 0.0263
I
Sensitivity (TPR)
1−Specificity (FPR)
TMC1
AUC: 0.748
CI: 0.691−0.806
J
TMC2
AUC: 0.609
CI: 0.552−0.666
K
TMC3
AUC: 0.658
CI: 0.602−0.714
L
TMC4
AUC: 0.903
CI: 0.870−0.936
M
TMC5
AUC: 0.616
CI: 0.564−0.668
N
TMC6
AUC: 0.673
CI: 0.611−0.736
O
TMC7
AUC: 0.768
CI: 0.720−0.816
P
TMC8
AUC: 0.878
CI: 0.834−0.922

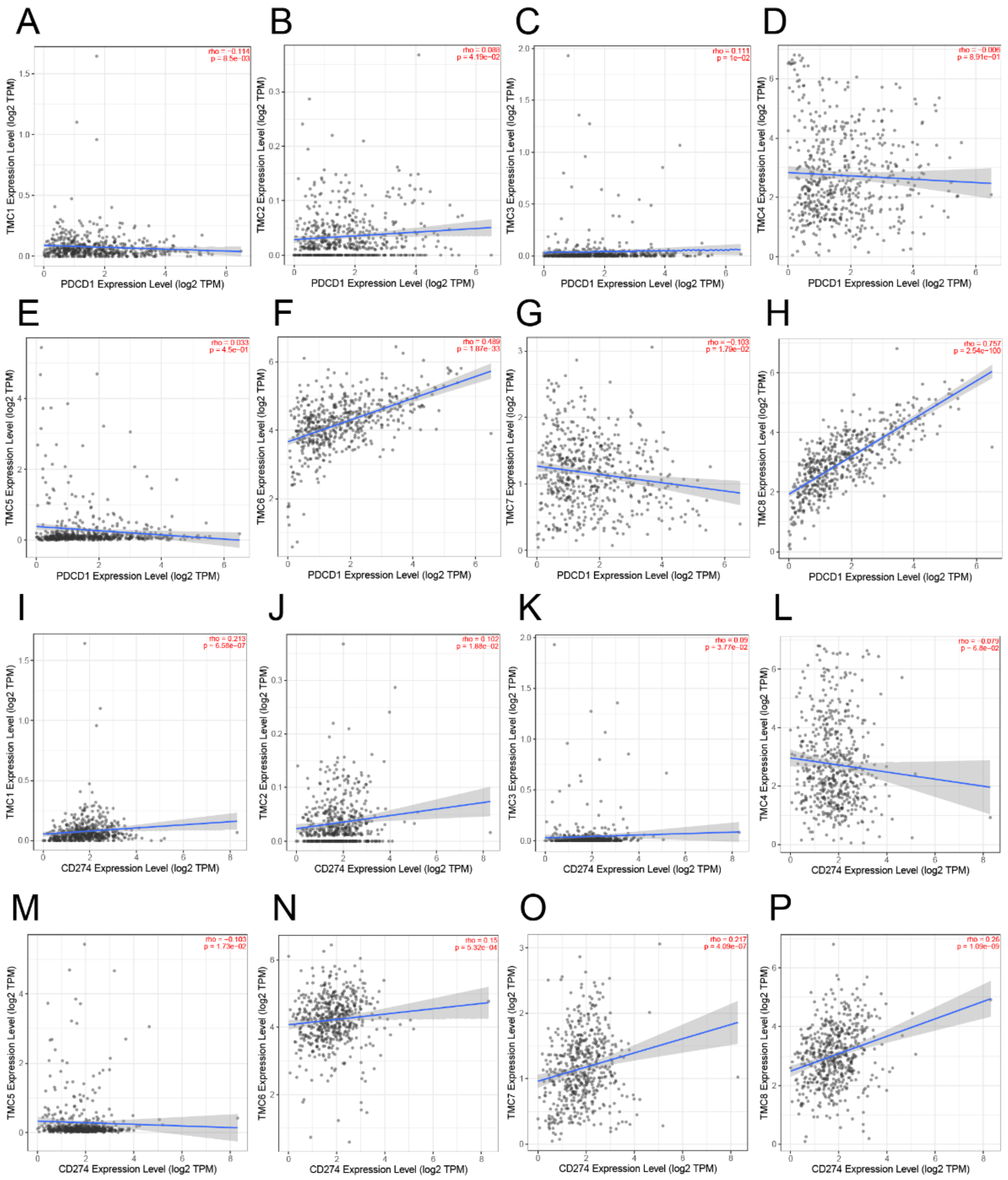
A
rho = −0.114
p = 8.5e−03
TMC1 Expression Level (log2 TPM)
PDCD1 Expression Level (log2 TPM)
B
rho = 0.088
p = 4.19e−02
TMC2 Expression Level (log2 TPM)
PDCD1 Expression Level (log2 TPM)
C
rho = 0.111
p = 1e−02
TMC3 Expression Level (log2 TPM)
PDCD1 Expression Level (log2 TPM)
D
rho = −0.006
p = 8.91e−01
TMC4 Expression Level (log2 TPM)
PDCD1 Expression Level (log2 TPM)
E
rho = 0.033
p = 4.5e−01
TMC5 Expression Level (log2 TPM)
PDCD1 Expression Level (log2 TPM)
F
rho = 0.489
p = 1.87e−33
TMC6 Expression Level (log2 TPM)
PDCD1 Expression Level (log2 TPM)
G
rho = −0.103
p = 1.79e−02
TMC7 Expression Level (log2 TPM)
PDCD1 Expression Level (log2 TPM)
H
rho = 0.757
p = 2.54e−100
TMC8 Expression Level (log2 TPM)
PDCD1 Expression Level (log2 TPM)
I
rho = 0.213
p = 6.58e−07
TMC1 Expression Level (log2 TPM)
CD274 Expression Level (log2 TPM)
J
rho = 0.102
p = 1.88e−02
TMC2 Expression Level (log2 TPM)
CD274 Expression Level (log2 TPM)
K
rho = 0.09
p = 3.77e−02
TMC3 Expression Level (log2 TPM)
CD274 Expression Level (log2 TPM)
L
rho = −0.079
p = 6.8e−02
TMC4 Expression Level (log2 TPM)
CD274 Expression Level (log2 TPM)
M
rho = −0.103
p = 1.73e−02
TMC5 Expression Level (log2 TPM)
CD274 Expression Level (log2 TPM)
N
rho = 0.15
p = 5.32e−04
TMC6 Expression Level (log2 TPM)
CD274 Expression Level (log2 TPM)
O
rho = 0.217
p = 4.09e−07
TMC7 Expression Level (log2 TPM)
CD274 Expression Level (log2 TPM)
P
rho = 0.26
p = 1.09e−09
TMC8 Expression Level (log2 TPM)
CD274 Expression Level (log2 TPM)

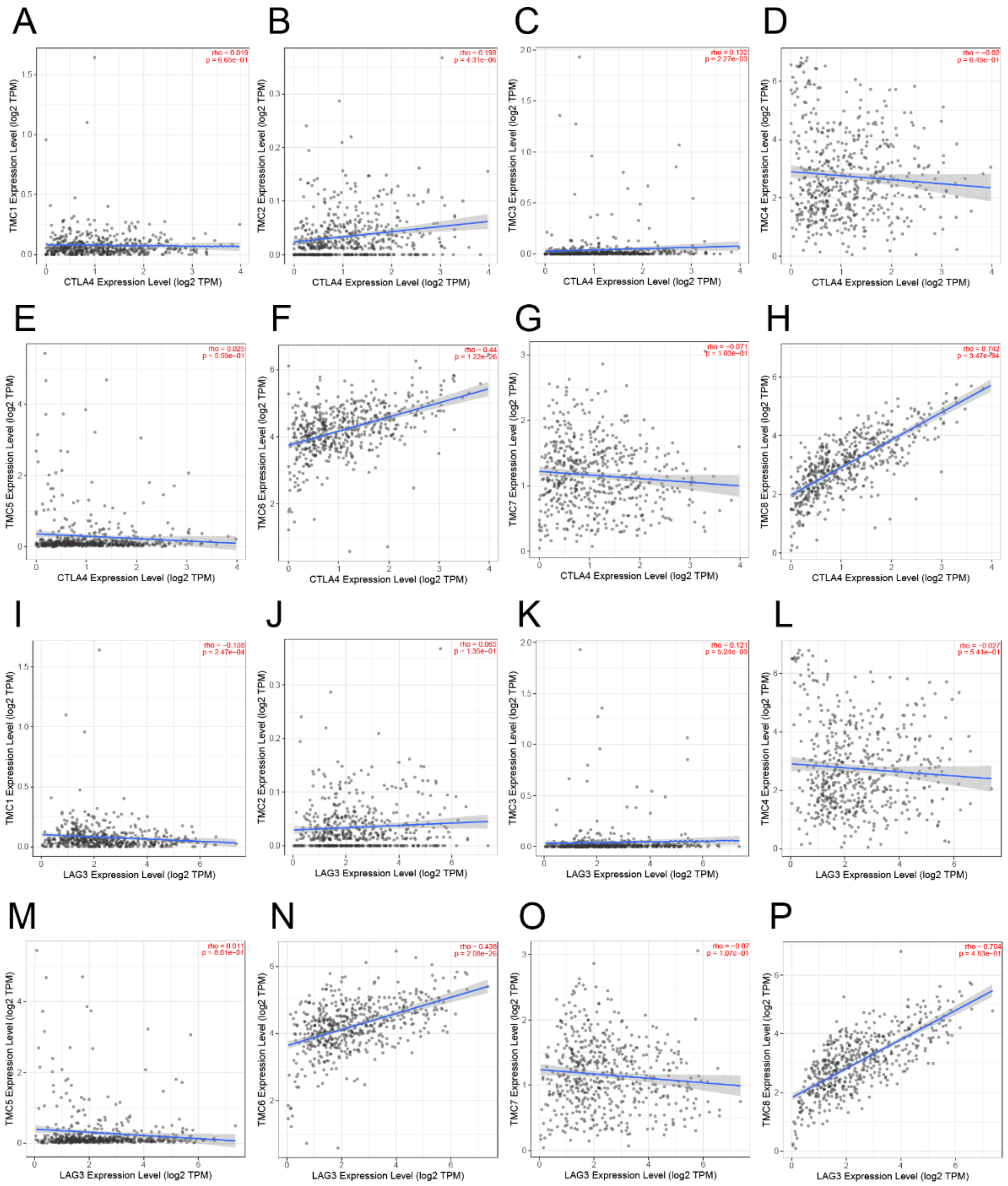
A
B
C
D
TMC1 Expression Level (log2 TPM)
TMC2 Expression Level (log2 TPM)
TMC3 Expression Level (log2 TPM)
TMC4 Expression Level (log2 TPM)
CTLA4 Expression Level (log2 TPM)
E
F
G
H
TMC5 Expression Level (log2 TPM)
TMC6 Expression Level (log2 TPM)
TMC7 Expression Level (log2 TPM)
TMC8 Expression Level (log2 TPM)
CTLA4 Expression Level (log2 TPM)
I
J
K
L
TMC1 Expression Level (log2 TPM)
TMC2 Expression Level (log2 TPM)
TMC3 Expression Level (log2 TPM)
TMC4 Expression Level (log2 TPM)
LAG3 Expression Level (log2 TPM)
M
N
O
P
TMC5 Expression Level (log2 TPM)
TMC6 Expression Level (log2 TPM)
TMC7 Expression Level (log2 TPM)
TMC8 Expression Level (log2 TPM)
LAG3 Expression Level (log2 TPM)

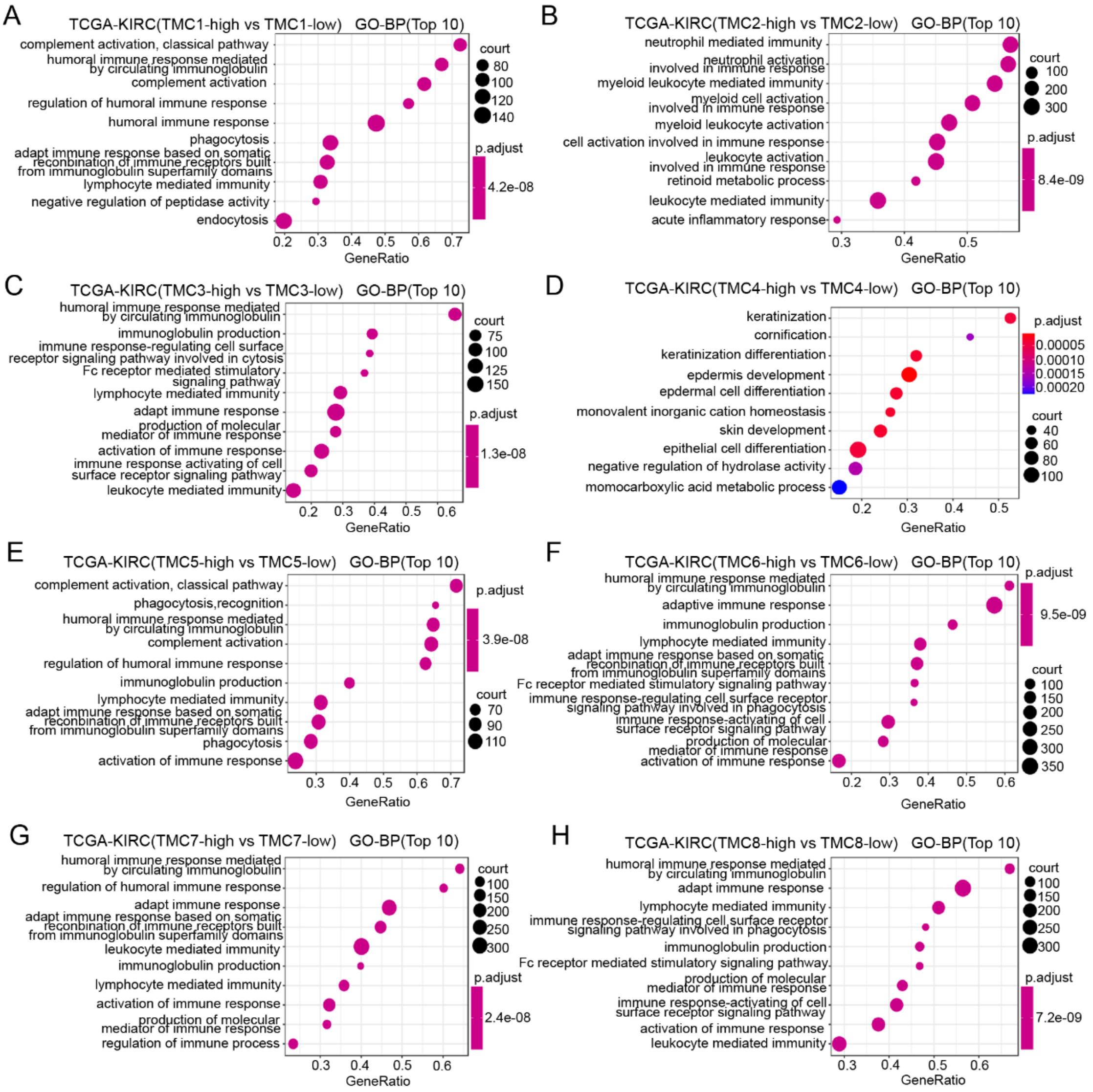
A
TCGA-KIRC(TMC1-high vs TMC1-low) GO-BP(Top 10)
complement activation, classical pathway
humoral immune response mediated by circulating immunoglobulin
complement activation
regulation of humoral immune response
humoral immune response
phagocytosis
adapt immune response based on somatic recombination of immune receptors built from immunoglobulin superfamily domains
lymphocyte mediated immunity
negative regulation of peptidase activity
endocytosis
0.2 0.3 0.4 0.5 0.6 0.7
GeneRatio
court
80
100
120
140
p.adjust
4.2e-08
B
TCGA-KIRC(TMC2-high vs TMC2-low) GO-BP(Top 10)
neutrophil mediated immunity
neutrophil activation involved in immune response
myeloid leukocyte mediated immunity
myeloid cell activation involved in immune response
myeloid leukocyte activation
cell activation involved in immune response
leukocyte activation involved in immune response
retinoid metabolic process
leukocyte mediated immunity
acute inflammatory response
0.3 0.4 0.5
GeneRatio
court
100
200
300
p.adjust
8.4e-09
C
TCGA-KIRC(TMC3-high vs TMC3-low) GO-BP(Top 10)
humoral immune response mediated by circulating immunoglobulin
immunoglobulin production
immune response-regulating cell surface receptor signaling pathway involved in cytosis
Fc receptor mediated stimulatory signaling pathway
lymphocyte mediated immunity
adapt immune response
production of molecular mediator of immune response
activation of immune response
immune response activating of cell surface receptor signaling pathway
leukocyte mediated immunity
0.2 0.3 0.4 0.5 0.6
GeneRatio
court
75
100
125
150
p.adjust
1.3e-08
D
TCGA-KIRC(TMC4-high vs TMC4-low) GO-BP(Top 10)
keratinization
cornification
keratinization differentiation
epdermis development
epdermal cell differentiation
monovalent inorganic cation homeostasis
skin development
epithelial cell differentiation
negative regulation of hydrolase activity
momocarboxylic acid metabolic process
0.2 0.3 0.4 0.5
GeneRatio
p.adjust
0.00005
0.00010
0.00015
0.00020
court
40
60
80
100
E
TCGA-KIRC(TMC5-high vs TMC5-low) GO-BP(Top 10)
complement activation, classical pathway
phagocytosis,recognition
humoral immune response mediated by circulating immunoglobulin
complement activation
regulation of humoral immune response
immunoglobulin production
lymphocyte mediated immunity
adapt immune response based on somatic recombination of immune receptors built from immunoglobulin superfamily domains
phagocytosis
activation of immune response
0.3 0.4 0.5 0.6 0.7
GeneRatio
p.adjust
3.9e-08
court
70
90
110
F
TCGA-KIRC(TMC6-high vs TMC6-low) GO-BP(Top 10)
humoral immune response mediated by circulating immunoglobulin
adaptive immune response
immunoglobulin production
lymphocyte mediated immunity
adapt immune response based on somatic recombination of immune receptors built from immunoglobulin superfamily domains
Fc receptor mediated stimulatory signaling pathway
immune response-regulating cell surface receptor signaling pathway involved in phagocytosis
immune response-activating of cell surface receptor signaling pathway
production of molecular mediator of immune response
activation of immune response
0.2 0.3 0.4 0.5 0.6
GeneRatio
p.adjust
9.5e-09
court
100
150
200
250
300
350
G
TCGA-KIRC(TMC7-high vs TMC7-low) GO-BP(Top 10)
humoral immune response mediated by circulating immunoglobulin
regulation of humoral immune response
adapt immune response
adapt immune response based on somatic recombination of immune receptors built from immunoglobulin superfamily domains
leukocyte mediated immunity
immunoglobulin production
lymphocyte mediated immunity
activation of immune response
production of molecular mediator of immune response
regulation of immune process
0.3 0.4 0.5 0.6
GeneRatio
court
100
150
200
250
300
p.adjust
2.4e-08
H
TCGA-KIRC(TMC8-high vs TMC8-low) GO-BP(Top 10)
humoral immune response mediated by circulating immunoglobulin
adapt immune response
lymphocyte mediated immunity
immune response-regulating cell surface receptor signaling pathway involved in phagocytosis
immunoglobulin production
Fc receptor mediated stimulatory signaling pathway
production of molecular mediator of immune response
immune response-activating of cell surface receptor signaling pathway
activation of immune response
leukocyte mediated immunity
0.3 0.4 0.5 0.6
GeneRatio
court
100
150
200
250
300
p.adjust
7.2e-09

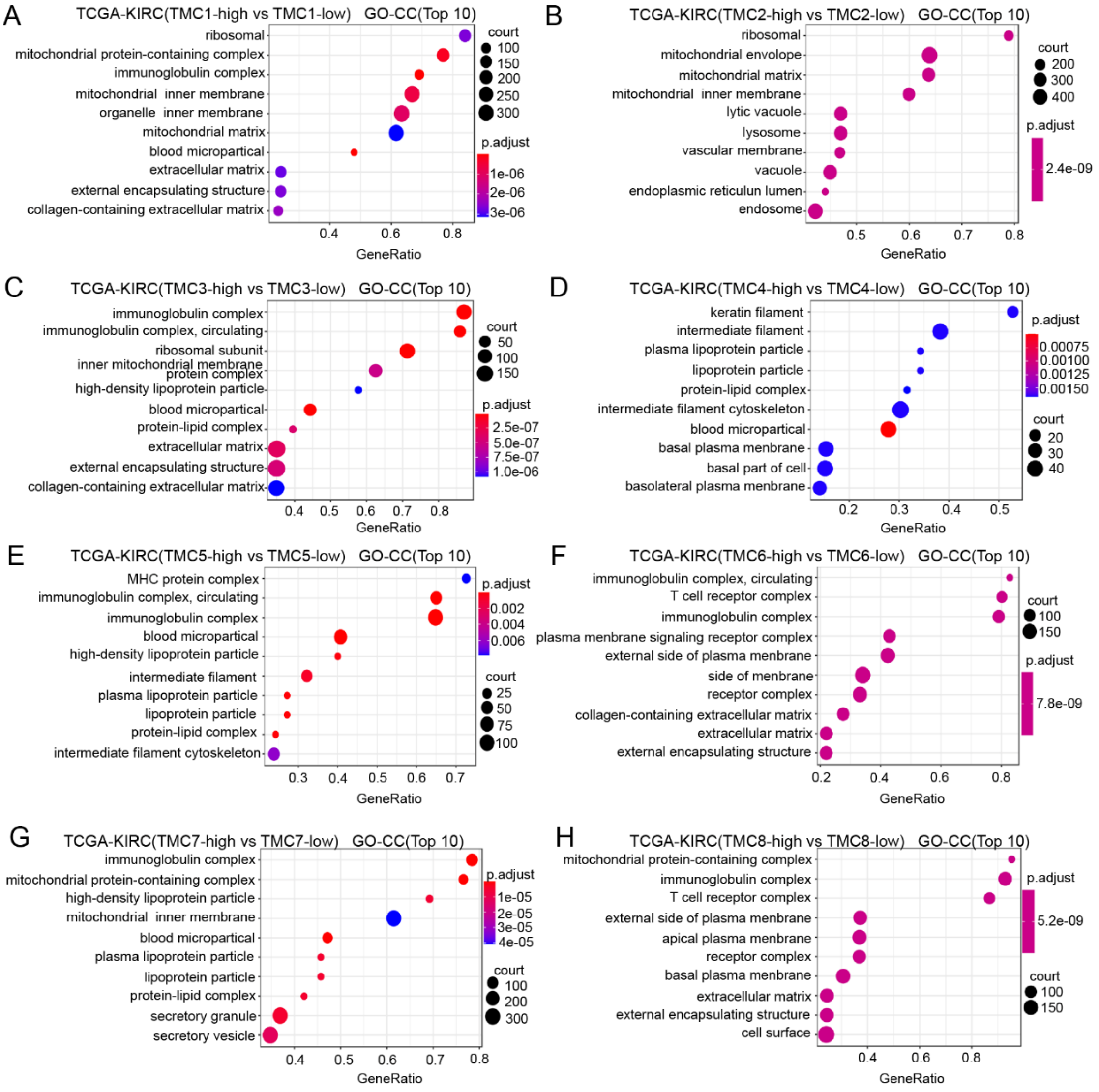
A
TCGA-KIRC(TMC1-high vs TMC1-low) GO-CC(Top 10)
ribosomal
mitochondrial protein-containing complex
immunoglobulin complex
mitochondrial inner membrane
organelle inner membrane
mitochondrial matrix
blood microparticle
extracellular matrix
external encapsulating structure
collagen-containing extracellular matrix
GeneRatio
court
p.adjust
B
TCGA-KIRC(TMC2-high vs TMC2-low) GO-CC(Top 10)
ribosomal
mitochondrial envelope
mitochondrial matrix
mitochondrial inner membrane
lytic vacuole
lysosome
vascular membrane
vacuole
endoplasmic reticulum lumen
endosome
GeneRatio
court
p.adjust
C
TCGA-KIRC(TMC3-high vs TMC3-low) GO-CC(Top 10)
immunoglobulin complex
immunoglobulin complex, circulating
ribosomal subunit
inner mitochondrial membrane protein complex
high-density lipoprotein particle
blood microparticle
protein-lipid complex
extracellular matrix
external encapsulating structure
collagen-containing extracellular matrix
GeneRatio
court
p.adjust
D
TCGA-KIRC(TMC4-high vs TMC4-low) GO-CC(Top 10)
keratin filament
intermediate filament
plasma lipoprotein particle
lipoprotein particle
protein-lipid complex
intermediate filament cytoskeleton
blood microparticle
basal plasma menbrane
basal part of cell
basolateral plasma menbrane
GeneRatio
p.adjust
court
E
TCGA-KIRC(TMC5-high vs TMC5-low) GO-CC(Top 10)
MHC protein complex
immunoglobulin complex, circulating
immunoglobulin complex
blood microparticle
high-density lipoprotein particle
intermediate filament
plasma lipoprotein particle
lipoprotein particle
protein-lipid complex
intermediate filament cytoskeleton
GeneRatio
p.adjust
court
F
TCGA-KIRC(TMC6-high vs TMC6-low) GO-CC(Top 10)
immunoglobulin complex, circulating
T cell receptor complex
immunoglobulin complex
plasma menbrane signaling receptor complex
external side of plasma menbrane
side of menbrane
receptor complex
collagen-containing extracellular matrix
extracellular matrix
external encapsulating structure
GeneRatio
court
p.adjust
G
TCGA-KIRC(TMC7-high vs TMC7-low) GO-CC(Top 10)
immunoglobulin complex
mitochondrial protein-containing complex
high-density lipoprotein particle
mitochondrial inner membrane
blood microparticle
plasma lipoprotein particle
lipoprotein particle
protein-lipid complex
secretory granule
secretory vesicle
GeneRatio
p.adjust
court
H
TCGA-KIRC(TMC8-high vs TMC8-low) GO-CC(Top 10)
mitochondrial protein-containing complex
immunoglobulin complex
T cell receptor complex
external side of plasma menbrane
apical plasma menbrane
receptor complex
basal plasma menbrane
extracellular matrix
external encapsulating structure
cell surface
GeneRatio
p.adjust
court

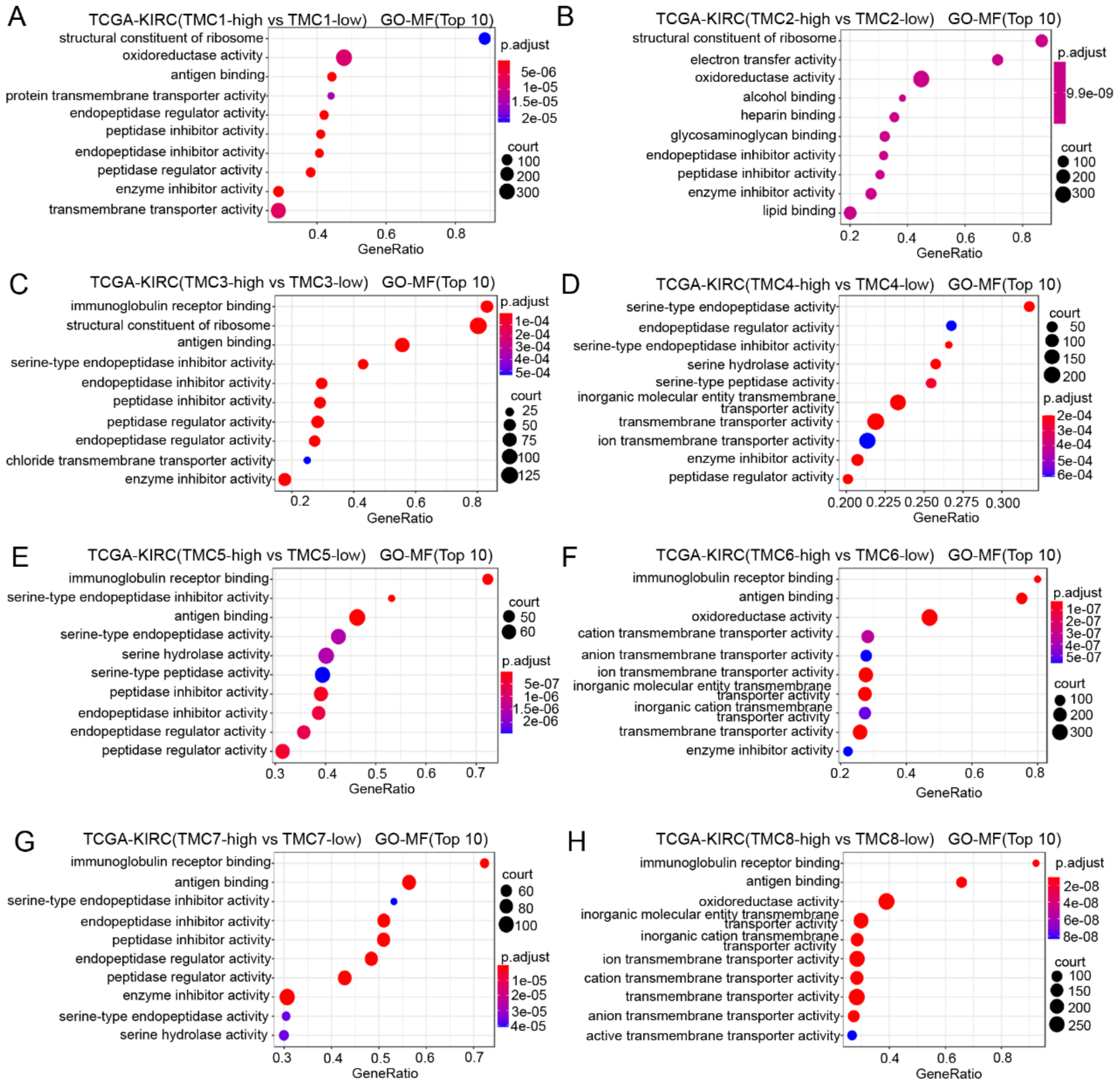

A
TCGA-KIRC(TMC1-high vs TMC1-low) GO-MF(Top 10)
structural constituent of ribosome
oxidoreductase activity
antigen binding
protein transmembrane transporter activity
endopeptidase regulator activity
peptidase inhibitor activity
endopeptidase inhibitor activity
peptidase regulator activity
enzyme inhibitor activity
transmembrane transporter activity
0.4 0.6 0.8
GeneRatio
p.adjust
5e-06
1e-05
1.5e-05
2e-05
court
100
200
300
B
TCGA-KIRC(TMC2-high vs TMC2-low) GO-MF(Top 10)
structural constituent of ribosome
electron transfer activity
oxidoreductase activity
alcohol binding
heparin binding
glycosaminoglycan binding
endopeptidase inhibitor activity
peptidase inhibitor activity
enzyme inhibitor activity
lipid binding
0.2 0.4 0.6 0.8
GeneRatio
p.adjust
9.9e-09
court
100
200
300
C
TCGA-KIRC(TMC3-high vs TMC3-low) GO-MF(Top 10)
immunoglobulin receptor binding
structural constituent of ribosome
antigen binding
serine-type endopeptidase inhibitor activity
endopeptidase inhibitor activity
peptidase inhibitor activity
peptidase regulator activity
endopeptidase regulator activity
chloride transmembrane transporter activity
enzyme inhibitor activity
0.2 0.4 0.6 0.8
GeneRatio
p.adjust
1e-04
2e-04
3e-04
4e-04
5e-04
court
25
50
75
100
125
D
TCGA-KIRC(TMC4-high vs TMC4-low) GO-MF(Top 10)
serine-type endopeptidase activity
endopeptidase regulator activity
serine-type endopeptidase inhibitor activity
serine hydrolase activity
serine-type peptidase activity
inorganic molecular entity transmembrane transporter activity
transmembrane transporter activity
ion transmembrane transporter activity
enzyme inhibitor activity
peptidase regulator activity
0.200 0.225 0.250 0.275 0.300
GeneRatio
court
50
100
150
200
p.adjust
2e-04
3e-04
4e-04
5e-04
6e-04
E
TCGA-KIRC(TMC5-high vs TMC5-low) GO-MF(Top 10)
immunoglobulin receptor binding
serine-type endopeptidase inhibitor activity
antigen binding
serine-type endopeptidase activity
serine hydrolase activity
serine-type peptidase activity
peptidase inhibitor activity
endopeptidase inhibitor activity
endopeptidase regulator activity
peptidase regulator activity
0.3 0.4 0.5 0.6 0.7
GeneRatio
court
50
60
p.adjust
5e-07
1e-06
1.5e-06
2e-06
F
TCGA-KIRC(TMC6-high vs TMC6-low) GO-MF(Top 10)
immunoglobulin receptor binding
antigen binding
oxidoreductase activity
cation transmembrane transporter activity
anion transmembrane transporter activity
ion transmembrane transporter activity
inorganic molecular entity transmembrane transporter activity
inorganic cation transmembrane transporter activity
transmembrane transporter activity
enzyme inhibitor activity
0.2 0.4 0.6 0.8
GeneRatio
p.adjust
1e-07
2e-07
3e-07
4e-07
5e-07
court
100
200
300
G
TCGA-KIRC(TMC7-high vs TMC7-low) GO-MF(Top 10)
immunoglobulin receptor binding
antigen binding
serine-type endopeptidase inhibitor activity
endopeptidase inhibitor activity
peptidase inhibitor activity
endopeptidase regulator activity
peptidase regulator activity
enzyme inhibitor activity
serine-type endopeptidase activity
serine hydrolase activity
0.3 0.4 0.5 0.6 0.7
GeneRatio
court
60
80
100
p.adjust
1e-05
2e-05
3e-05
4e-05
H
TCGA-KIRC(TMC8-high vs TMC8-low) GO-MF(Top 10)
immunoglobulin receptor binding
antigen binding
oxidoreductase activity
inorganic molecular entity transmembrane transporter activity
inorganic cation transmembrane transporter activity
ion transmembrane transporter activity
cation transmembrane transporter activity
transmembrane transporter activity
anion transmembrane transporter activity
active transmembrane transporter activity
0.4 0.6 0.8
GeneRatio
p.adjust
2e-08
4e-08
6e-08
8e-08
court
100
150
200
250

Supplementary Figure legends

Figure S1:

The mRNA level of TMCs in RCCC tissues. *$p < 0.05$, **$p < 0.01$, ***$p < 0.001$, ns: no statistically significant. (A-H) The mRNA expression of TMCs in RCCC from the GEPIA database. Tumor tissues are shown in red, and normal tissues are shown in gray. (I-P) The mRNA expression of TMCs in RCCC from the UALCAN database.

Figure S2:

Effect of TMC family expression on survival of RCCC patients. (A-H) Association between TMC family expression and RFS of RCCC patients. (I-P) Association between TMC family expression and PFI of RCCC patients.

Figure S3:

Correlation analysis between TMC family expression and clinical stage of RCCC patients. (A–K) represents TMC genes TMC1–8. The graphs were generated using the GEPIA. (I-P) ROC curve of TMCs showing their diagnostic capability for RCCC.

Figure S4:

Correlation of TMC family expression and the level of PDCD1 and CD274. (A-H) Associations between TMC expression and the level of PDCD1. (I-P) Associations between TMC expression and the level of CD274. The scatter plots were generated using the TIMER 2.0 database.

Figure S5:

Correlation of TMC family expression and the level of CTLA4 and LAG3. (A-H) Associations between TMC expression and the level of CTLA4. (I-P) Associations between TMC expression and the level of LAG3. The scatter plots were generated using the TIMER 2.0 database.

Figure S6:

The GO-BP functional enrichment analysis of the TMCs based on TCGA-KIRC cohort. (A-H) represents TMC genes TMC1-8.

Figure S7:

The GO-CC functional enrichment analysis of the TMCs based on TCGA-KIRC cohort. (A-H) represents TMC genes TMC1-8.

Figure S8:

The GO-MF functional enrichment analysis of the TMCs based on TCGA-KIRC cohort. (A-H) represents TMC genes TMC1-8.
