# Supplementary figures and images for: Comprehensive analysis of the prognosis and immune infiltration of TMC family members in renal clear cell carcinoma
Source: Sci Rep. 2023 Jul 19;13:11668. doi: 10.1038/s41598-023-38914-z (PMC10356759; doi:10.1038/s41598-023-38914-z)

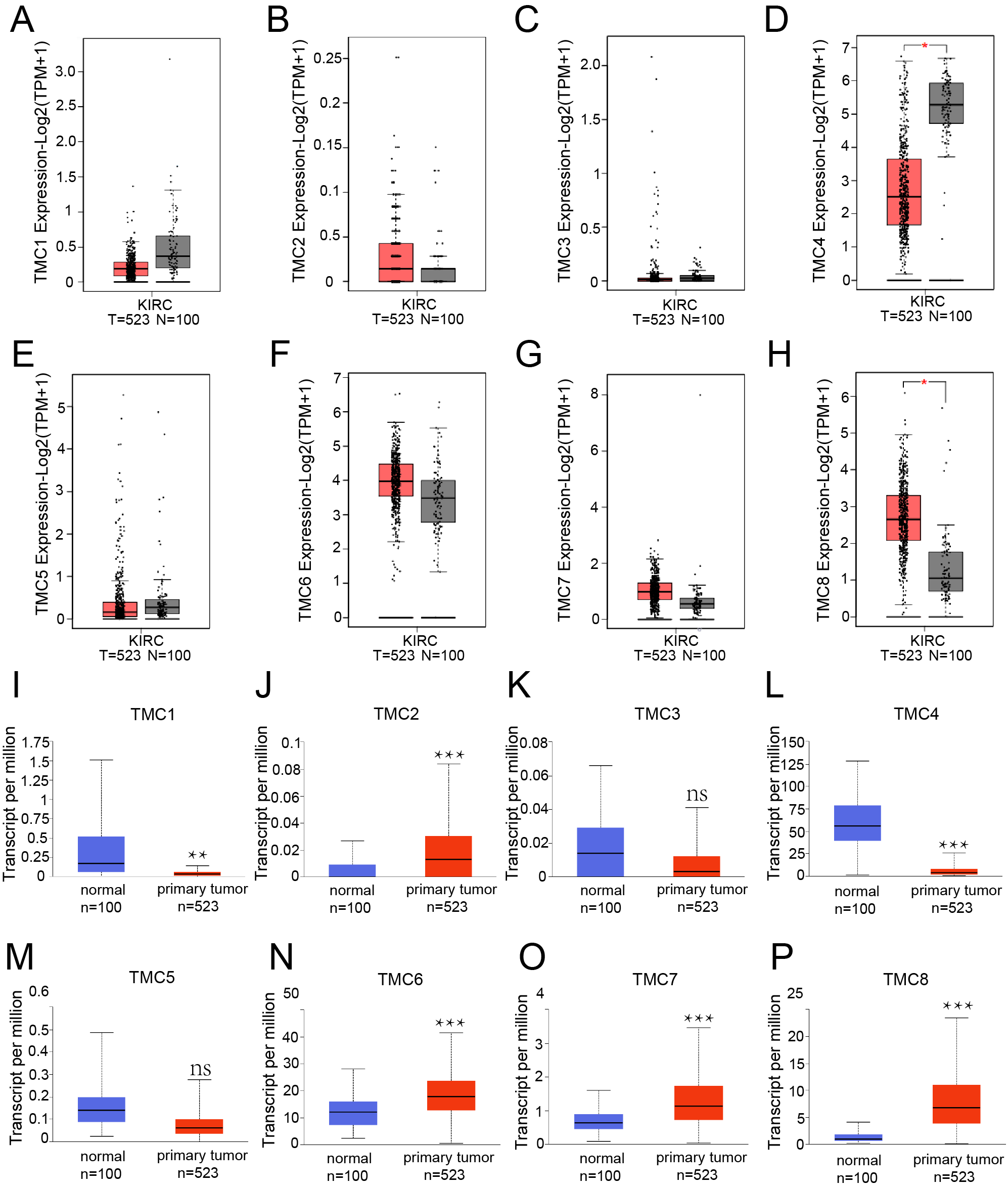

Supplement: Supplementary file 1 — Supplementary Information. [file 41598_2023_38914_MOESM1_ESM.zip › supplementary material/figureS1.tif]

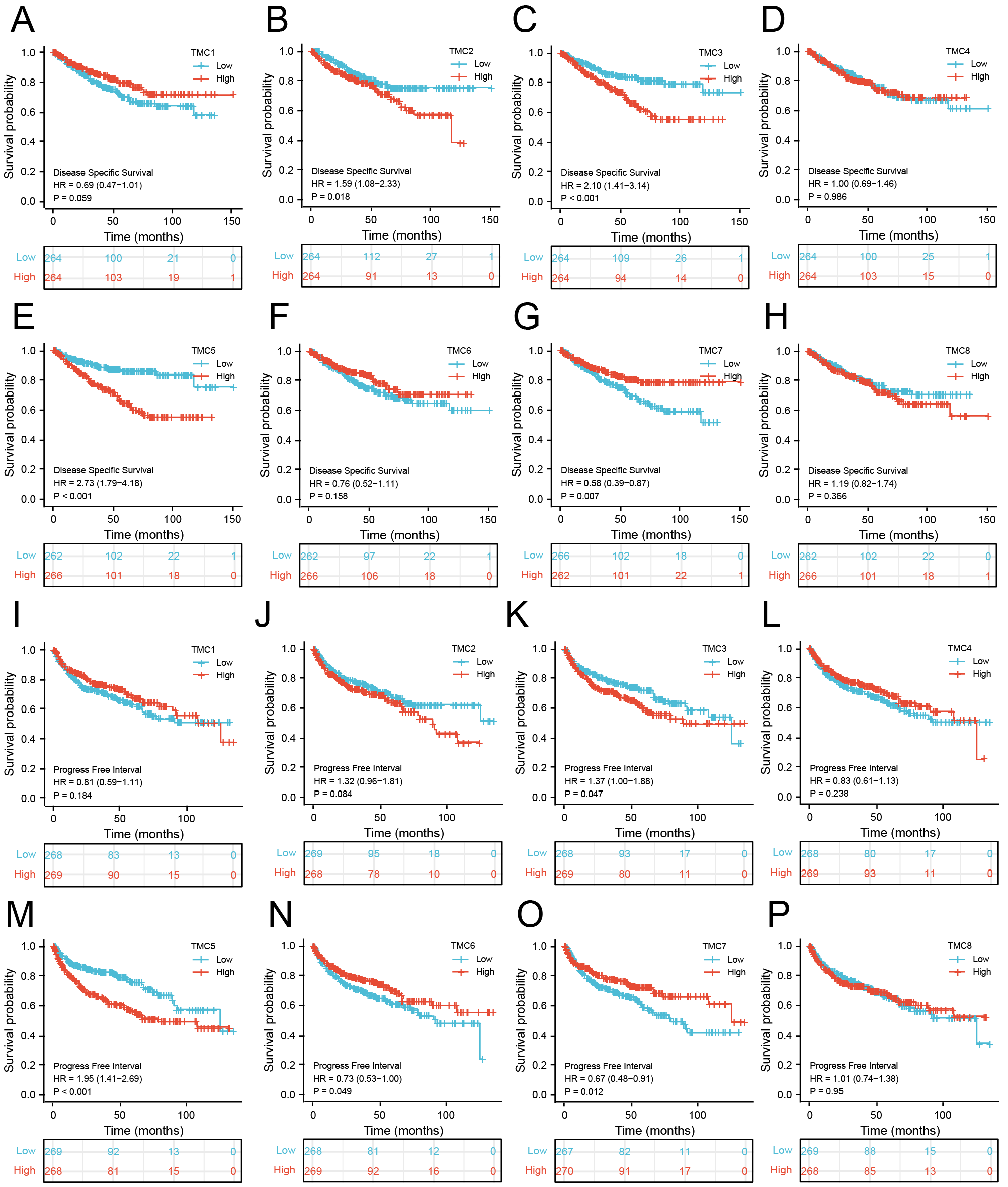

Supplement: Supplementary file 1 — Supplementary Information. [file 41598_2023_38914_MOESM1_ESM.zip › supplementary material/figureS2.tif]

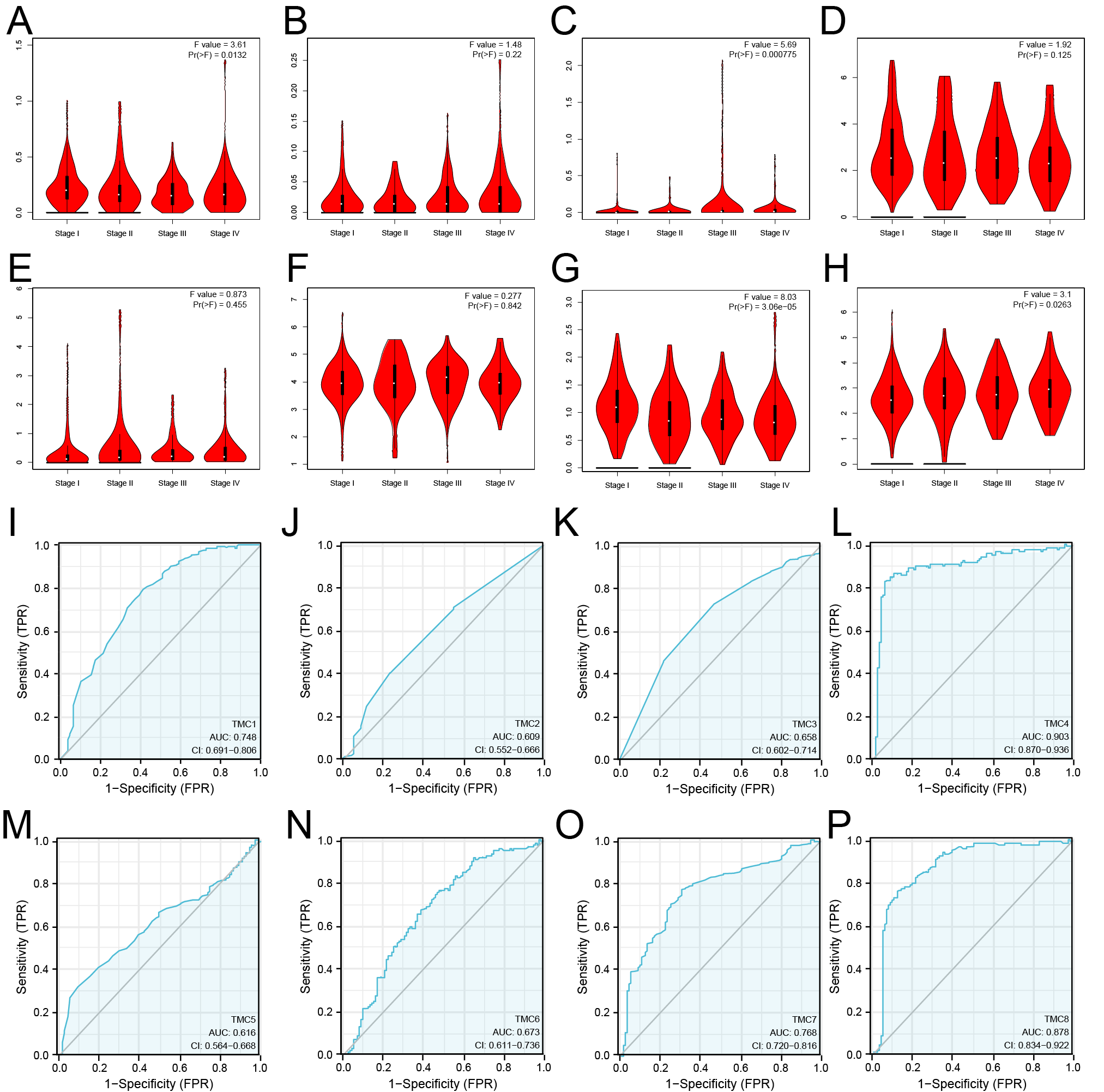

Supplement: Supplementary file 1 — Supplementary Information. [file 41598_2023_38914_MOESM1_ESM.zip › supplementary material/figureS3.tif]

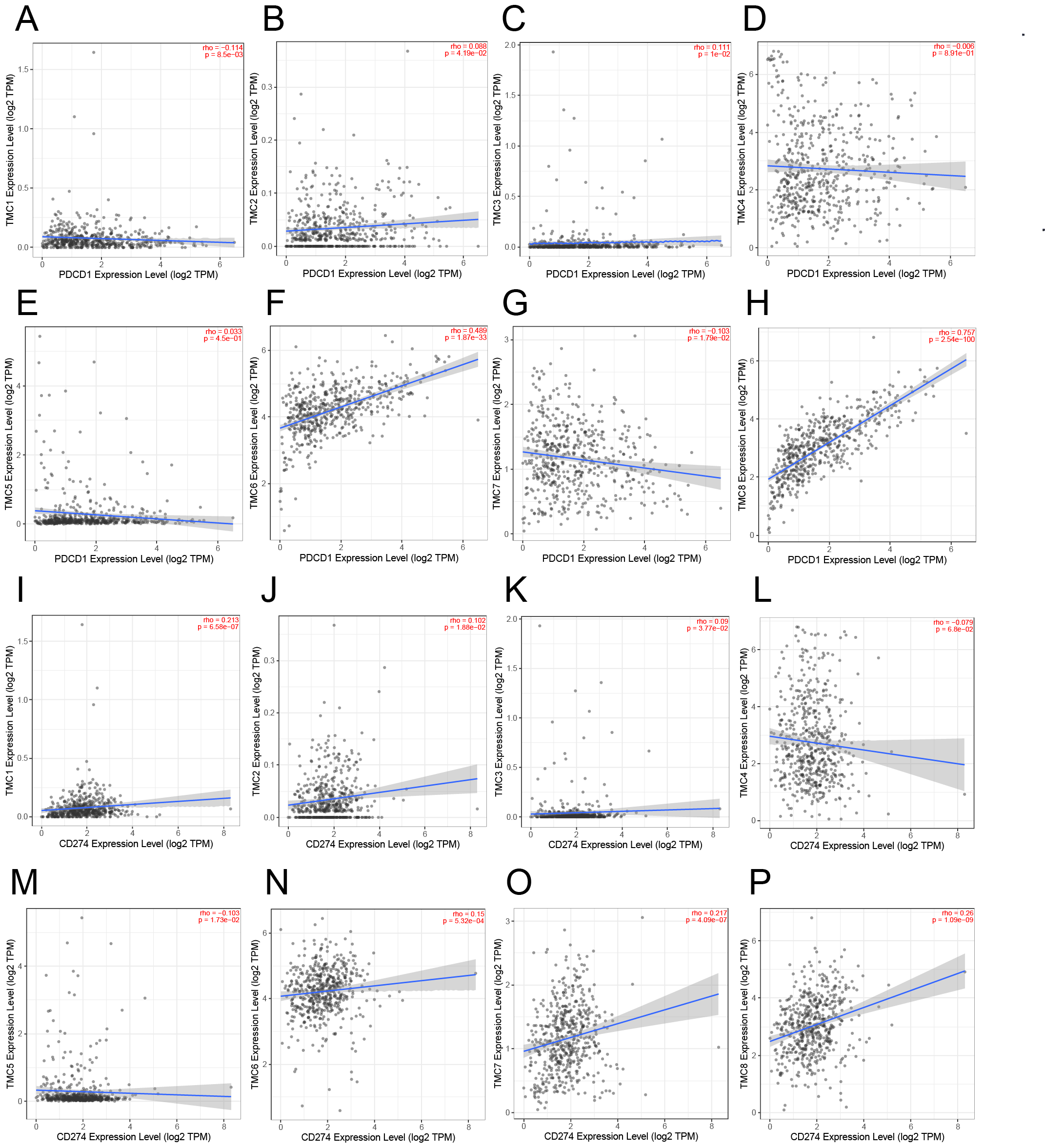

Supplement: Supplementary file 1 — Supplementary Information. [file 41598_2023_38914_MOESM1_ESM.zip › supplementary material/figureS4.tif]

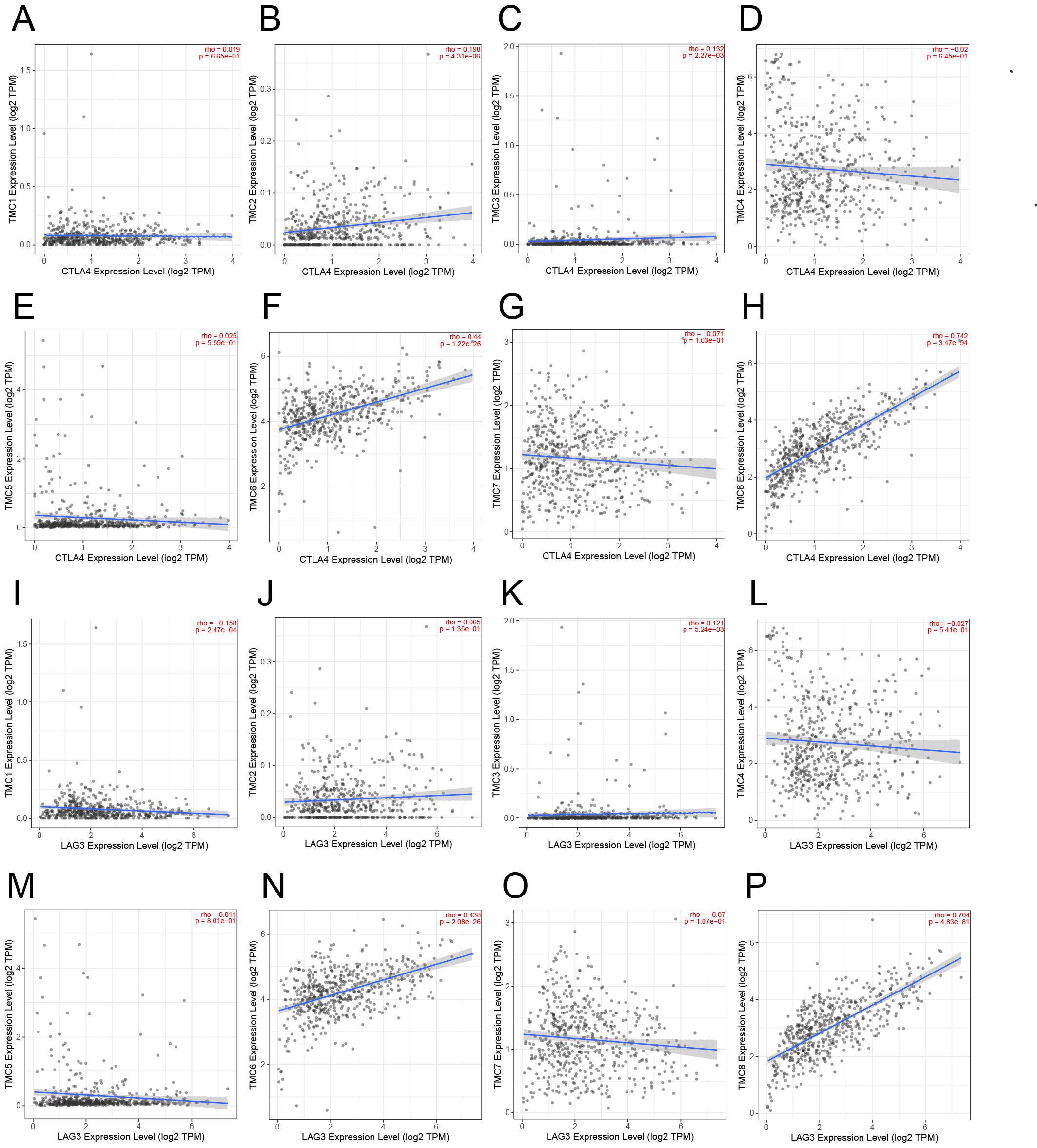

Supplement: Supplementary file 1 — Supplementary Information. [file 41598_2023_38914_MOESM1_ESM.zip › supplementary material/figureS5.tif]

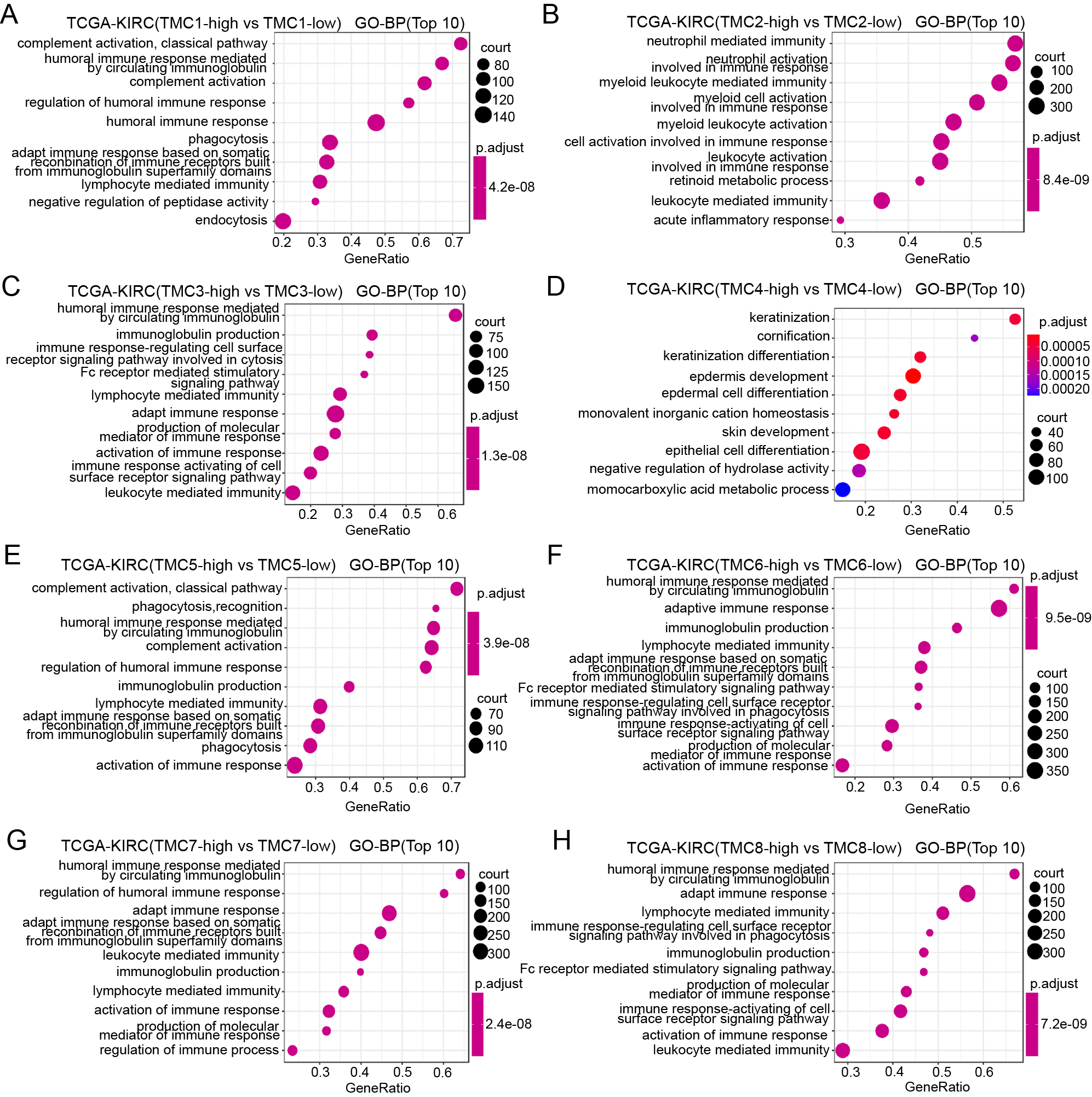

Supplement: Supplementary file 1 — Supplementary Information. [file 41598_2023_38914_MOESM1_ESM.zip › supplementary material/figureS6.tif]

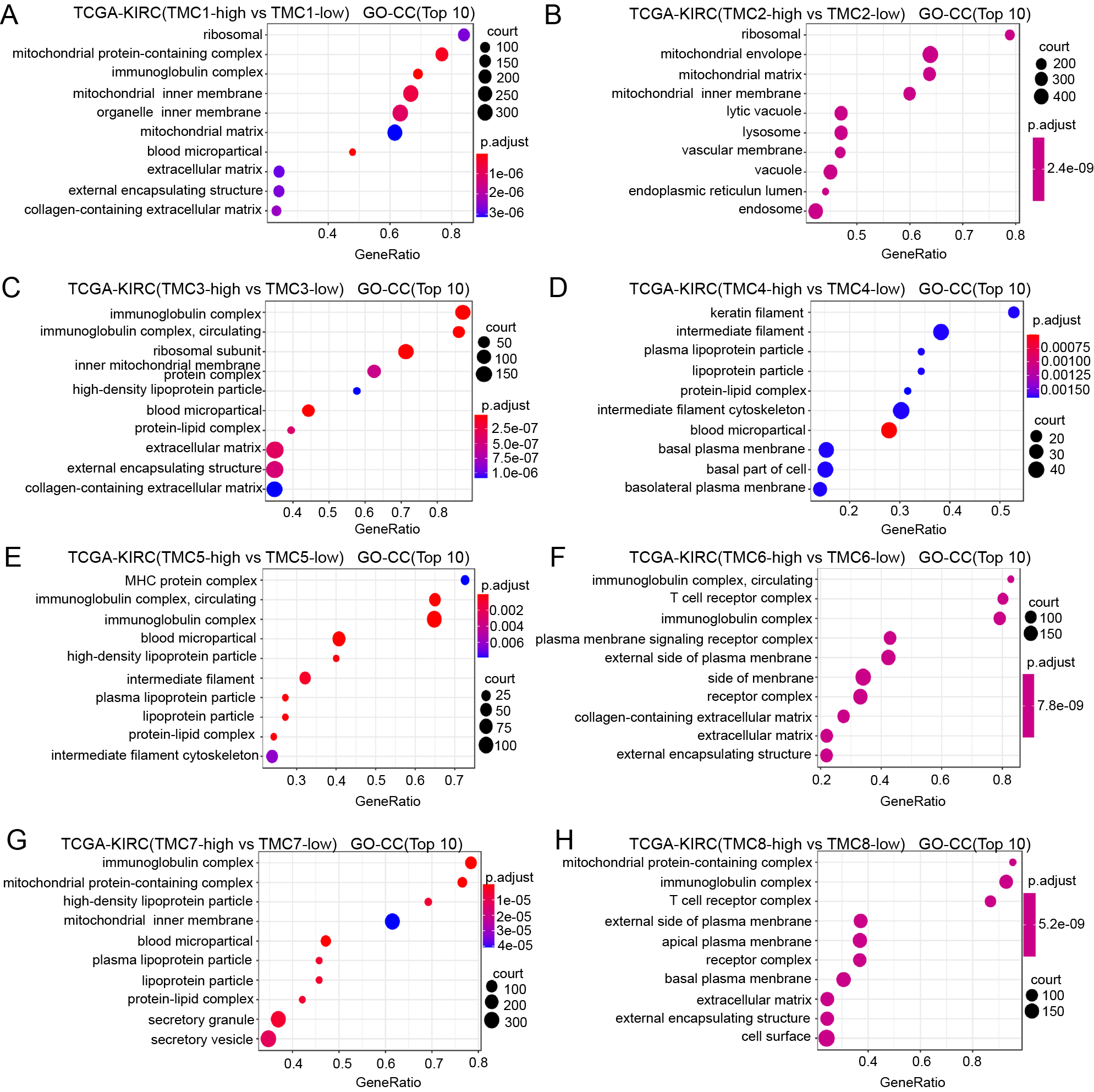

Supplement: Supplementary file 1 — Supplementary Information. [file 41598_2023_38914_MOESM1_ESM.zip › supplementary material/figureS7.tif]

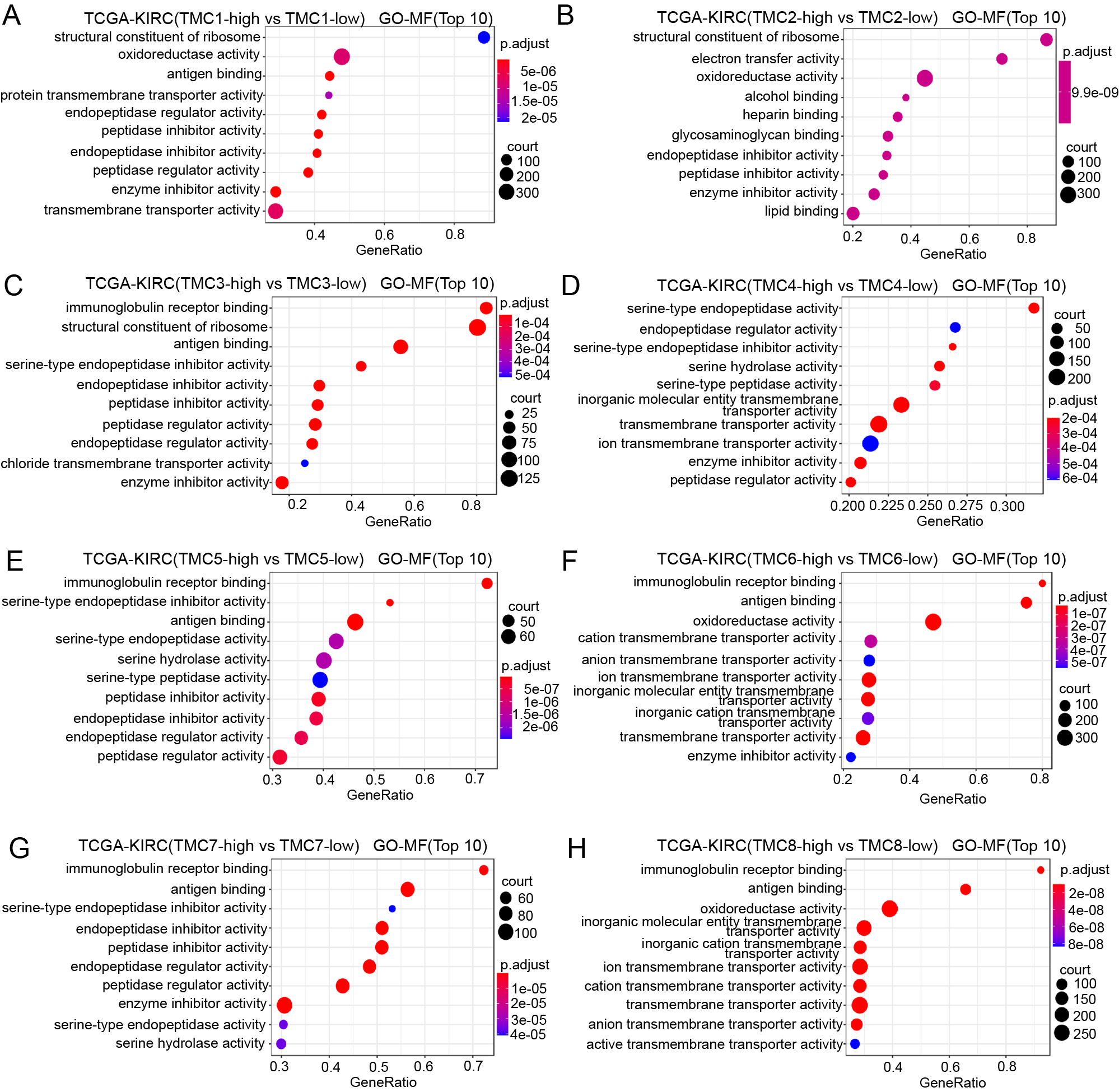

Supplement: Supplementary file 1 — Supplementary Information. [file 41598_2023_38914_MOESM1_ESM.zip › supplementary material/figureS8.tif]
